# A Computer Simulation Approach to Assessing Therapeutic Intervention Points for the Prevention of Cytokine-Induced Cartilage Breakdown

**DOI:** 10.1002/art.38297

**Published:** 2014-03-28

**Authors:** CJ Proctor, C Macdonald, JM Milner, AD Rowan, TE Cawston

**Affiliations:** 1 MRC–Arthritis Research UK Centre for Integrated Research into Musculoskeletal Ageing and Newcastle UniversityNewcastle upon Tyne, UK; 2 Newcastle UniversityNewcastle upon Tyne, UK

## Abstract

**Objective:**

To use a novel computational approach to examine the molecular pathways involved in cartilage breakdown and to use computer simulation to test possible interventions for reducing collagen release.

**Methods:**

We constructed a computational model of the relevant molecular pathways using the Systems Biology Markup Language, a computer-readable format of a biochemical network. The model was constructed using our experimental data showing that interleukin-1 (IL-1) and oncostatin M (OSM) act synergistically to up-regulate collagenase protein levels and activity and initiate cartilage collagen breakdown. Simulations were performed using the COPASI software package.

**Results:**

The model predicted that simulated inhibition of JNK or p38 MAPK, and overexpression of tissue inhibitor of metalloproteinases 3 (TIMP-3) led to a reduction in collagen release. Overexpression of TIMP-1 was much less effective than that of TIMP-3 and led to a delay, rather than a reduction, in collagen release. Simulated interventions of receptor antagonists and inhibition of JAK-1, the first kinase in the OSM pathway, were ineffective. So, importantly, the model predicts that it is more effective to intervene at targets that are downstream, such as the JNK pathway, rather than those that are close to the cytokine signal. In vitro experiments confirmed the effectiveness of JNK inhibition.

**Conclusion:**

Our study shows the value of computer modeling as a tool for examining possible interventions by which to reduce cartilage collagen breakdown. The model predicts that interventions that either prevent transcription or inhibit the activity of collagenases are promising strategies and should be investigated further in an experimental setting.

Rheumatoid arthritis and osteoarthritis are both characterized by loss of extracellular matrix (ECM) in the cartilage of articular joints. Cartilage is maintained by chondrocytes that secrete ECM components, such as collagen and aggrecan. In both diseases, joint damage occurs as the cartilage matrix is destroyed by proteinases that are up-regulated by a variety of different stimuli. While ADAMTS-4 and ADAMTS-5 are mainly responsible for the degradation of aggrecan, collagen is degraded by the collagenases (matrix metalloproteinase 1 [MMP-1] and MMP-13). Tissue inhibitor of metalloproteinases (TIMPs) are endogenous inhibitors of MMPs, and TIMP-3 can also inhibit ADAMTS (1,2). Aggrecan breakdown is reversible, but the irreversibility of collagen release makes its prevention key for developing effective therapies for arthritis. This requires detailed knowledge of the mechanisms involved in collagen breakdown. We have previously used cell and organ systems to examine the pathways that lead to the up-regulation of the collagenases following the addition of cytokines to chondrocytes (3–5). Since collagenases are initially synthesized in an inactive form, they require activators to be present in order to effect collagen release (6).

In our in vitro models, we have used combinations of interleukin-1 (IL-1) and oncostatin M (OSM) to promote cartilage collagen breakdown; neither cytokine alone reproducibly leads to collagen cleavage (5–7). IL-1 is a proinflammatory cytokine that binds to the IL-1 receptor (IL-1R) and recruits IL-1R–associated kinase (IRAK) proteins, which are phosphorylated. This leads to recruitment of tumor necrosis factor receptor–associated factor 6 (TRAF6) proteins, which phosphorylate JNK. Activated JNK then phosphorylates c-Jun, which forms homodimers or binds c-Fos to form heterodimers, which form part of the activator protein 1 (AP-1) transcription factor. The c-Jun homodimers have low affinity for DNA (8), whereas AP-1, which is composed of c-Fos and c-Jun, has high affinity for the promoter regions of many target genes, such as MMPs, phosphatases, ADAMTS, and the transcription factor Sp-1. Sp-1 inhibits TIMP-1 transcription by binding to a repressive element in the first intron of TIMP-1 (9). Messenger RNA (mRNA) for c-Fos has a very short half-life, is not expressed under normal cellular conditions, and is only weakly expressed after stimulation with IL-1. Therefore, IL-1 stimulation alone will favor the formation of c-Jun homodimers, leading to lower levels of up-regulation of AP-1 target genes than those with IL-1 plus OSM stimulation.

OSM has antiinflammatory and proinflammatory roles, with signaling primarily via the JAK/STAT pathway (10). There is evidence that p38 phosphorylates c-Fos to enhance its transcriptional activity (11). OSM synergizes with IL-1 to increase the expression of MMPs in chondrocytes (12), and since STAT proteins do not bind MMP promoters in chondrocytes, this synergy occurs through STAT stimulation of c-Fos expression, leading to changes in AP-1 composition that regulate MMP expression. It should be noted that c-Fos is regulated at the transcriptional level, whereas c-Jun is regulated post-translationally via phosphorylation. The pathways involved in collagen release are thus complex, involving cross-talk between different pathways and many feedback loops.

It has become increasingly recognized that systems modeling approaches are required to complement experimental work, and computational models have been widely used in the fields of cancer, cardiovascular diseases, and neurodegeneration; to date, this approach is not established in the study of musculoskeletal diseases (13–15). In this study, we used our existing in vitro data from cell and organ culture models to construct an in silico model of cartilage collagen breakdown following stimulation with IL-1 and OSM combinations. Our aim was to demonstrate how computational models can be developed using current knowledge of the system to highlight important gaps in knowledge, to test new hypotheses, and to predict outcomes for different therapeutic approaches.

## METHODS

### Model construction

The model contains 3 separate submodels: the first describes the IL-1/JNK signaling pathway, the second describes the OSM/STAT-3 signaling pathway, and the third describes proMMP activation and the release of aggrecan and collagen (see Supplementary Figures 1–3, available on the *Arthritis & Rheumatology* web site at http://onlinelibrary.wiley.com/doi/10.1002/art.38297/abstract). This modular approach allows new components to be easily added and allows existing modules to be reused in further models. The models were encoded in the Systems Biology Markup Language (SBML), a computer-readable format for representing biochemical networks (16), using the Python tool SBML-shorthand (17). The integrated model was deposited in the BioModels Database (18) and assigned the identifier MODEL1305280001. The main features and assumptions of the model are described below. Full details of the model species, parameters, and reactions are given in Supplementary Tables 1–3 (available on the *Arthritis & Rheumatology* web site at http://onlinelibrary.wiley.com/doi/10.1002/art.38297/abstract).

### IL-1 signaling pathway module

This module contains details of the signaling pathway from IL-1 binding to its receptor, leading to a cascade of phosphorylation events via IRAK-2, TRAF6, JNK, p38, and finally c-Jun. We assumed that phosphorylated c-Jun can form dimers that have low transcriptional activity for the following target genes: c-Jun, MMP-1, MMP-3, MMP-13, ADAMTS-4, protein phosphatase 4 (PP-4), dual-specificity protein phosphatase 16 (DUSP-16), and MAPK phosphatase 1 (MKP-1). We also included basal transcription of c-Jun, TIMP-1, and TIMP-3, which does not involve c-Jun. For species with available experimental data for both the mRNA and protein levels, we included transcription and translation reactions (c-Jun, MMPs, and ADAMTS-4); otherwise, we modeled protein synthesis as one reaction (DUSP-16, PP-4, and MKP-1).

### OSM signaling pathway module

This module contains details of the signaling pathway from OSM binding to its receptor (OSMR), leading to phosphorylation of JAK-1 and STAT-3. We assumed that pSTAT-3 can be transported to the nucleus, where it may transcribe c-Fos, receptor-type protein tyrosine phosphatase T (PTPRT), and suppressor of cytokine signaling 3 (SOCS-3). If pSTAT-3 is dephosphorylated in the nucleus, it is transported back to the cytoplasm. SOCS-3 binds to OSMR in competition with OSM and so provides a negative feedback loop by which to stop this signaling pathway. The c-Fos protein is phosphorylated by p38 and dephosphorylated by either DUSP-16 or an unspecified phosphatase. Phosphorylated c-Fos can reversibly bind to phosphorylated c-Jun to form a complex, which represents the AP-1 transcription factor. We include transcription of the following genes by c-Fos/c-Jun heterodimers: c-Fos, c-Jun, MMP-1, MMP-3, MMP-13, ADAMTS-4, DUSP-16, PP-4, MKP-1, Sp-1, TIMP-1, and TIMP-3. PP-4 binds to TRAF6 to prevent the binding of TRAF6 to IRAK-2 and so provides an additional negative feedback loop to stop OSM signaling.

### Activation of proMMPs and degradation of aggrecan and collagen module

We assumed that proMMP-1 and proMMP-3 are activated by a generic activator (MMP activator) and that MMP-3 also activates proMMP-1 and proMMP-13. Initially, aggrecan is bound to collagen and behaves as a complex, since it has been shown that aggrecan protects collagen from degradation. We modeled aggrecan degradation by ADAMTS-4, which results in an unprotected collagen molecule. Free collagen can then be degraded by MMP-1 or MMP-13. We assumed that MMP-13 has greater activity at cleaving collagen than MMP-1 but that there are much higher levels of MMP-1 compared to MMP-13, which means that both collagenases are important for collagen release (19). We also assumed that activated pools of MMPs are removed with a half-life of about 30 hours. It has been shown that TIMP-1 is more effective at inhibiting MMPs whereas TIMP-3 mainly inhibits ADAMTS (1), and this assumption is included in our model.

### Parameter values

**Figure 1:**
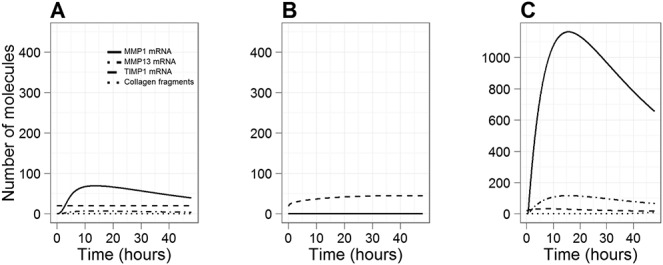
Simulation results showing the effect of interleukin-1 (IL-1) and/or oncostatin M (OSM) on the expression of matrix metalloproteinase 1 (MMP-1), MMP-13, and tissue inhibitor of metalloproteinases 1 (TIMP-1), using a simulated time period of 48 hours. Curves show the level of MMP-1 mRNA, MMP-13 mRNA, TIMP-1 mRNA, and collagen fragments. A, Effect of IL-1 alone. B, Effect of OSM alone. C, Effect of IL-1 plus OSM. Note that in B, MMP-1, MMP-13, and collagen levels are all zero; thus, the individual lines are not visible.

Parameters for the signaling pathways were chosen to fit experimental data where available. For example, we used published data on receptor binding affinities (20,21), time course data on kinase activity (22,23), data on phosphorylation and localization of STAT-3 (23), and time course data on SOCS-3 expression (24). We used data from our laboratory to fit the kinetics of c-Fos induction and the return of c-Fos to basal levels (see Supplementary Figure 4, available on the *Arthritis & Rheumatology* web site at http://onlinelibrary.wiley.com/doi/10.1002/art.38297/abstract). As we wished to use stochastic as well as deterministic simulations, we needed to use low numbers of molecules so that multiple simulations could be performed in reasonable time frames. We therefore used values in the region of 0–200 for basal expression, with levels increasing to ∼500–30,000 after cytokine-induced up-regulation. In a deterministic simulation, the level of the mRNA or protein remains fixed under basal conditions, but in a stochastic simulation, the levels may fluctuate around the mean value (compare Supplementary Figure 5 [available on the *Arthritis & Rheumatology* web site at http://online library.wiley.com/doi/10.1002/art.38297/abstract] with Figure 1). The level of MMP-1 (mRNA) is often 10-fold higher than that of MMP-13 (25). Thus, assuming equal degradation rates for MMP-1 and MMP-13 mRNA, we set the transcription rate of MMP-1 at 10 times higher than that of MMP-13.

### Model validation

The model was validated using experimental data that were not used in construction of the model (26,27). The details are given in Supplementary Figure 9 and Supplementary Table 7 (available on the *Arthritis & Rheumatology* web site at http://onlinelibrary.wiley.com/doi/10.1002/art.38297/abstract).

### Simulated treatments and interventions

The integrated model was used to assess possible therapeutic interventions, since cartilage breakdown does not occur in any of the individual submodels. We mimicked the addition of cytokines and MMP activator by altering the initial values of IL-1, OSM, and/or MMP activator before running the simulations (see Supplementary Table 4). Details of the species and parameters used to simulate possible interventions are shown in Supplementary Table 5 (Supplementary Tables 4 and 5 available on the *Arthritis & Rheumatology* web site at http://onlinelibrary.wiley.com/doi/10.1002/art.38297/abstract).

### Modeling tools

We carried out all the deterministic simulations using the COPASI software tool (28). Stochastic simulations were carried out on a computer cluster using the Gillespie algorithm (direct method) (29) and code developed by staff members of Newcastle University (30). We analyzed the model output using the R statistical programming language, and created the graphs using the R package ggplot2 (31). Network figures were constructed in CellDesigner (32) using the standard Systems Biology Graphical Notation (SBGN) (33).

### Previous experimental data used for constructing model

Human T/C-28a4 chondrocytes were stimulated with IL-1α (1 ng/ml), OSM (10 ng/ml), or IL-1α (1 ng/ml) plus OSM (10 ng/ml). The test reagents were added to the medium at the start of the experiment, and cells were harvested at different time points thereafter (4, 8, 12, 24, 48, and 72 hours) (4).

## RESULTS

### IL-1 and OSM have synergistic effects on MMP-1, MMP-13, and TIMP-1 expression and collagen release

Simulations were performed for 48 hours (virtual time) to examine the kinetics of MMP-1, MMP-13, and TIMP-1 induction (Figure 1). No collagen release occurred at this time point. In the model, addition of IL-1 alone led to an increase in MMP-1 expression, which peaked at ∼12 hours, but there was no induction of TIMP-1 above basal levels (Figure 1A). The addition of OSM alone led to an early induction of TIMP-1, which peaked at ∼2–4 hours and then slowly returned to basal levels; however, there was no induction of MMP-1 or collagen release (Figure 1B).

Modeling the addition of IL-1 and OSM together led to a synergistic induction of MMP-1, which peaked at ∼8 hours, with levels being ∼20-fold higher than those seen with IL-1 alone (Figure 1C). There was also a lower induction of TIMP-1 than was modeled with OSM alone. The simulation output showed that the level of MMP-13 was ∼10 times lower than that of MMP-1 throughout the time course, as demonstrated experimentally (25). These results compare well to the experimental data whereby human T/C-28a4 chondrocytes were stimulated with IL-1α, OSM, or IL-1α plus OSM (see Methods for details) (4). Therefore, our model assumption that c-Jun homodimers have very low transcriptional activity compared to c-Fos/c-Jun heterodimers produces results that are consistent with the experimental data.

### Simulated addition of MMP activator leads to collagen release in the presence of IL-1 plus OSM

**Figure 2:**
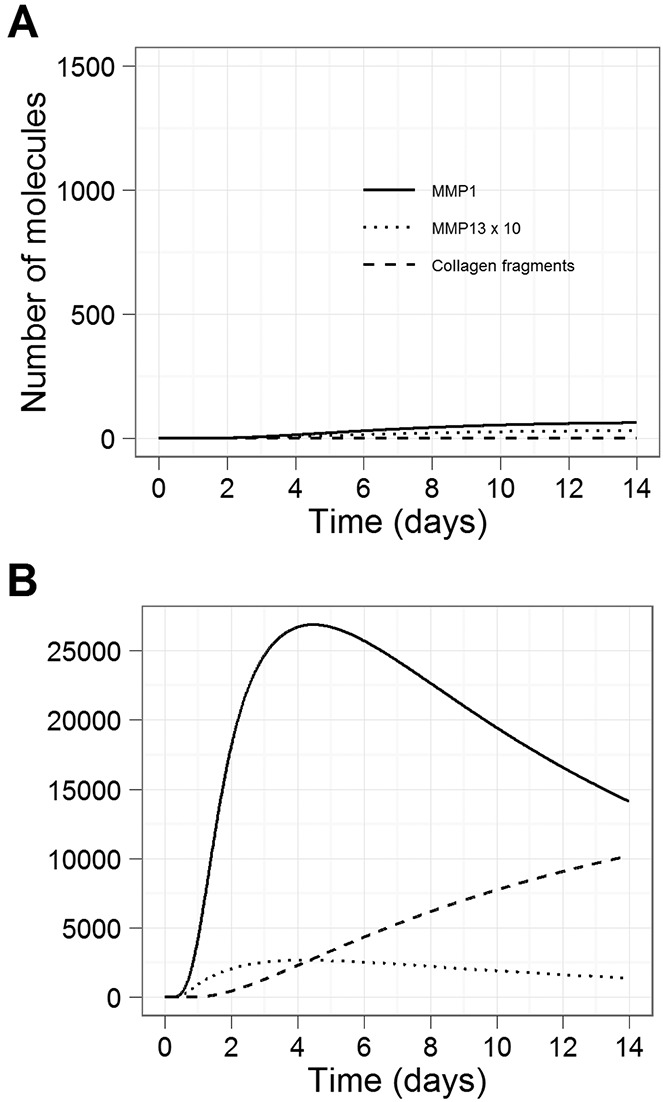
Simulation results showing the effect of an MMP-activating protease on the activation of MMPs and on collagen release, using a simulated time period of 14 days. Curves show the levels of active MMP-1 protein, active MMP-13 protein (scaled by a factor of 10), and collagen fragments. In each simulation, both IL-1 and OSM were added. A, Effect without MMP activator. B, Effect with addition of MMP activator. See Figure 1 for definitions.

Further simulations were performed over a 14-day period to examine the kinetics of collagen release with and without the addition of MMP activator (Figure 2). In the absence of MMP activator, the model output showed that ∼0.03% of collagen was released (by MMPs) by day 14 due to low basal levels of MMP activator following the addition of IL-1 plus OSM (Figure 2A). However, aggrecan was cleaved by ADAMTS-4, with ∼87% released by day 14. If an activator of collagenases was added at the start of the simulation, then proMMPs were processed at a much greater rate, and by day 14, 10% of collagen was released in the model (Figure 2B). The rate of aggrecan release was not affected by the addition of MMP activator.

### The model was used to assess therapeutic intervention points for the prevention of cytokine-induced cartilage breakdown

In order to assess the effects of potential therapeutic interventions, we used the model with IL-1 plus OSM and high levels of activating protease, such that collagen release occurred. We ran simulations for a time period of 14 days and compared the levels of aggrecan and collagen fragments with different simulated doses of each treatment/addition.

#### IL-1 receptor antagonist is predicted to have only limited beneficial effects

**Figure 3:**
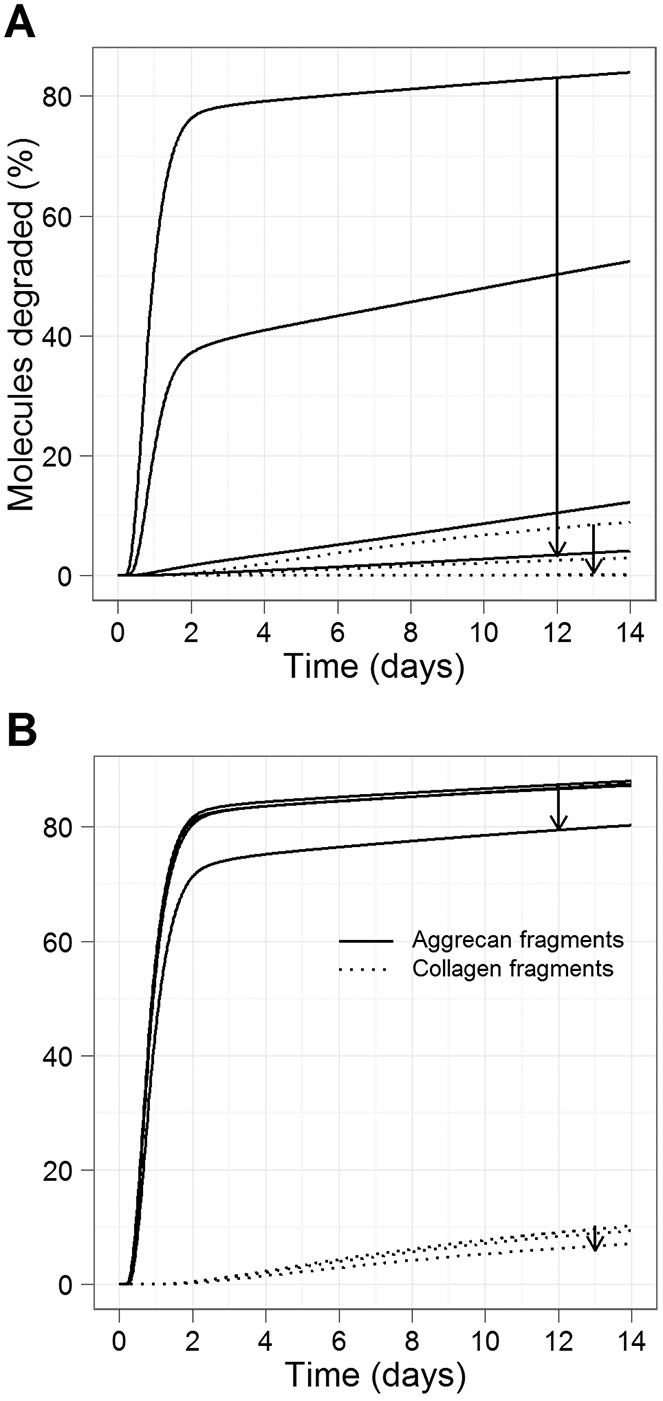
Simulation results for interventions with interleukin-1 receptor (IL-1R) or oncostatin M receptor (OSMR) antagonists, using a simulated time period of 14 days. Simulated conditions consisted of IL-1 plus OSM plus matrix metalloproteinase activator. Curves show the percentage of aggrecan and collagen degraded. A, Effect of IL-1R antagonist. B, Effect of OSMR antagonist. Arrows in A and B show the direction of increase in the ratio of receptor antagonist to receptor (1, 10, 100, 1,000).

The model predicted that very high levels of IL-1R antagonist (IL-Ra) would need to be added for there to be any significant effect on collagen release (100 times more antagonist than IL-1 receptors). This is because even low levels of IL-1R binding to IL-1 could initiate signaling, which eventually led to downstream events that culminated in collagen release. Thus, the model predicted that this intervention may delay or slow down collagen release, but cannot prevent initiation of the disease process. It is therefore likely that such an intervention used alone would not be very beneficial (Figure 3A).

#### OSMR antagonist is predicted to be less effective than IL-1 receptor antagonist

The model predicted that the addition of OSMR antagonists would be much less effective than IL-1R antagonists (Figure 3B). It was necessary to increase the ratio of OSMR antagonist by 10^7^ to have the same effect as a ratio of 10^3^ for IL-1Ra. This is because even low levels of OSMR binding to OSM can lead to the synergistic effect of IL-1 and OSM. The simultaneous addition of IL-1 and OSMR antagonists at the same concentration did not significantly reduce the levels of aggrecan and collagen fragments as compared to using IL-1Ra alone (see Supplementary Table 6, available on the *Arthritis & Rheumatology* web site at http://onlinelibrary.wiley.com/doi/10.1002/art.38297/abstract).

#### JAK-1 inhibition is ineffective as an intervention to reduce collagen release

**Figure 4:**
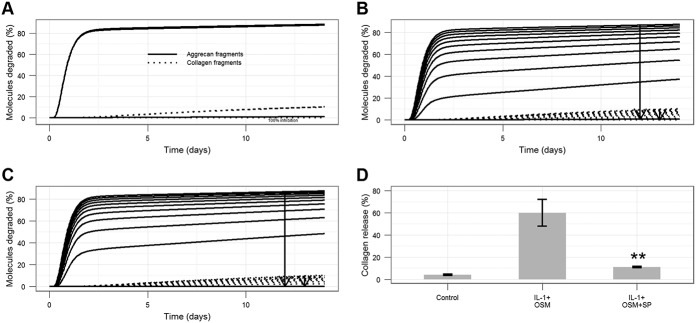
Simulation results showing the effect of inhibiting JAK-1, JNK, or p38 activity, using a simulated time period of 14 days. Simulated conditions consisted of IL-1 plus OSM plus MMP activator. Curves show the percentage of aggrecan and collagen degraded. A, Effect of JAK-1 inhibition. B, Effect of p38 inhibition. Arrows show the direction of increase (0–100%, in steps of 10; *k*_*phoscFos*_ = 5 × 10^−7^, 4.5 × 10^−7^, 4 × 10^−7^, …, 0) molecules^−1^ seconds^−1^). C, Effect of JNK inhibition. Arrows show the direction of increase (0–100%, in steps of 10; *k*_*phoscJun*_ = 1 × 10^−4^, 9 × 10^−5^, 8 × 10^−5^, …, 0 molecules^−1^ seconds^−1^). D, Effect of JNK inhibition on IL-1 plus OSM–induced cartilage breakdown. Bovine nasal cartilage discs were cultured in the presence of IL-1 (0.5 ng/ml) plus OSM (10 ng/ml) in the presence or absence of the JNK inhibitor SP600125 (SP; 30 μ*M*) or DMSO control. Medium was removed on day 7, and fresh reagents added. On day 14, the medium was removed, and the remaining cartilage was papain digested. The hydroxyproline assay was used to measure the release of collagen into the medium on day 7 and day 14. Values are the mean ± SEM of data accumulated from a minimum of 2 different experiments of a total of 4 experiments conducted. ∗∗ = *P* < 0.01 for cytokine treatment versus cytokine plus inhibitor treatment, by *t*-test. See Figure 1 for definitions.

In the model, JAK-1 phosphorylated only STAT-3, and so to simulate inhibition of this kinase, we varied the value of the parameter for STAT-3 phosphorylation (*k*_*phosSTAT3*_) from 0 (100% inhibition) to 0.005 (no inhibition). The effect of 100% inhibition was marked by a reduction in collagen release, from ∼10% to <1% on day 14, and a reduction in aggrecan release, from 87.5% to ∼1.0% (Figure 4A). However, 90% inhibition did not lead to any significant reduction in collagen release, and even 98% inhibition only reduced collagen fragments by <1%.

#### Inhibition of p38 or JNK produces a moderate reduction in collagen release

**Figure 5:**
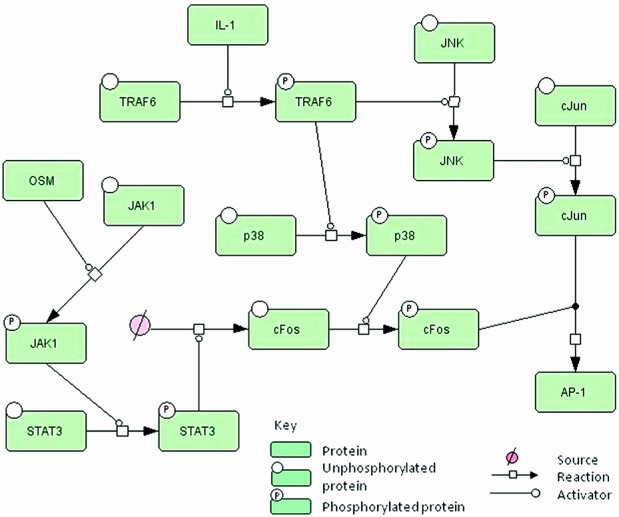
Simplified network diagram showing the involvement of JNK and p38 in the system. Interleukin-1 (IL-1) activates tumor necrosis factor receptor–associated factor 6 (TRAF6), which phosphorylates both p38 and JNK. JNK phosphorylates c-Jun, and p38 phosphorylates c-Fos, which has been up-regulated via the oncostatin M (OSM)/JAK-1/STAT-3 signaling pathway. Phosphorylated c-Fos binds to phosphorylated c-Jun to form the activator protein 1 (AP-1) complex. See Supplementary Figures 1 and 2 for diagrams showing all of the reactions (available on the *Arthritis & Rheumatology* web site at http://onlinelibrary.wiley.com/doi/10.1002/art.38297/abstract).

We simulated the inhibition of p38. In our model, p38 was induced by IL-1 due to phosphorylation by IRAK-2/TRAF6; however, it then acted in the OSM/STAT pathway by subsequently phosphorylating c-Fos, which bound phosphorylated c-Jun to form an AP-1 complex (Figure 5). We found that the model predicted that p38 inhibition had a much greater effect on collagen release than did JAK-1 inhibition (Figure 4B). Previously published data, which were not used in the model construction, demonstrated that p38 inhibition is effective at blocking IL-1–stimulated cartilage collagen release (34). JNK is phosphorylated by IRAK-2/TRAF6, and phosphorylated JNK subsequently phosphorylates c-Jun. The model predicted that inhibition of JNK, by reducing the rate of *k*_*phoscFos*_ from 0 to 100%, led to a reduction in collagen release (Figure 4C), and similar to p38, this was a more effective intervention than JAK-1 inhibition (Figure 4A). In order to confirm this prediction, we conducted an in vitro experiment in which we cultured cytokine-stimulated cartilage in the presence of a JNK inhibitor, and showed that cartilage collagen release was almost completely blocked by the JNK inhibitor SP600125 (Figure 4D).

#### Overexpression of TIMP-3 has a greater beneficial effect than overexpression of TIMP-1

**Figure 6:**
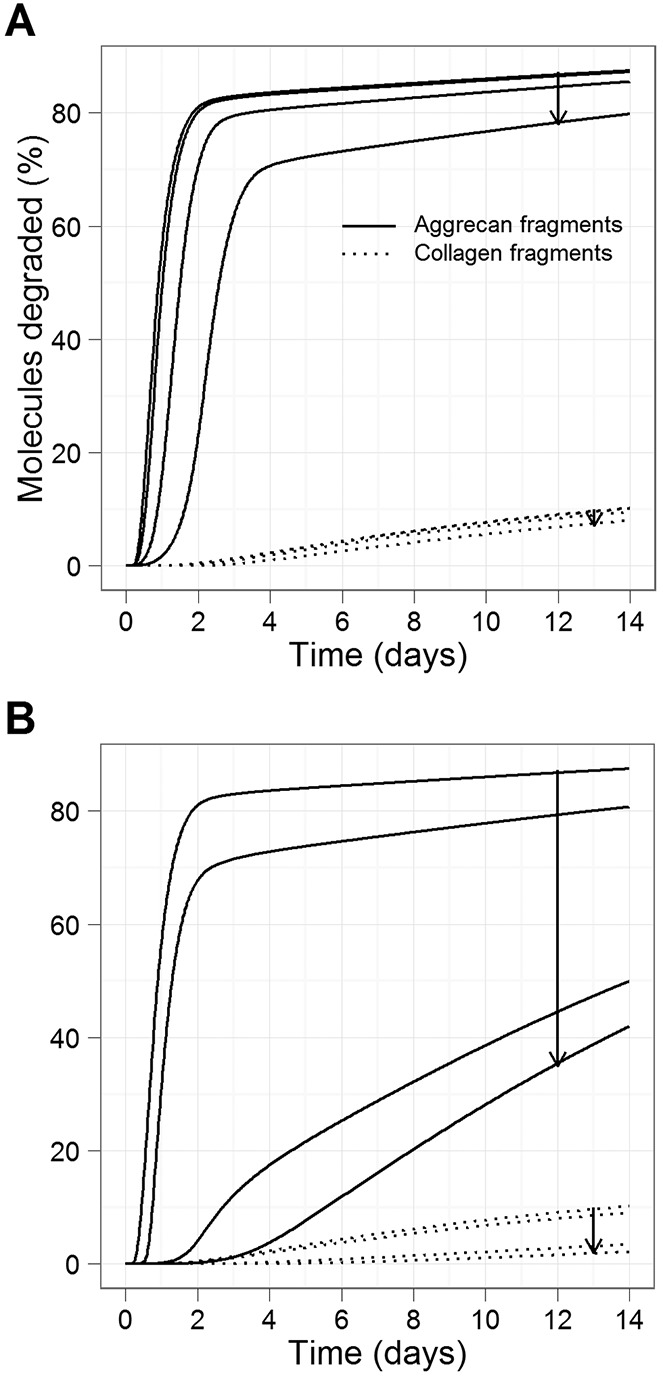
Simulation results showing model predictions for the overexpression of TIMP-1 or TIMP-3 protein, using a simulated time period of 14 days. Simulated conditions consisted of IL-1 plus OSM plus MMP activator. Curves show the percentage of aggrecan and collagen degraded. A, Effect of TIMP-1 overexpression. B, Effect of TIMP-3 overexpression. Arrows in A and B show the direction of increase (2 × 10^2^, 2 × 10^3^, 2 × 10^4^, 2 × 10^5^ molecules). See Figure 1 for definitions.

To simulate overexpression of TIMP-1 or TIMP-3, we varied the initial amount of protein from 200 to 200,000 molecules, with 3 intervals on a logarithmic scale (Figure 6). TIMP-1 overexpression delayed the onset of aggrecan and collagen release but only slightly reduced the maximum amount of degradation by day 14 (Figure 6A). Interestingly, TIMP-3 overexpression led to a much greater delay in aggrecan release and decreased the amount of collagen release by day 14 (Figure 6B).

### Stochastic effects are important and have implications for treatments

It is known that stochastic effects are important in biologic systems, may partly explain the cellular heterogeneity observed in experimental systems, and similarly account for the observed differences in loss of cartilage collagen in patients, in that some patients experience rapid joint destruction while others have relatively slow progression. Factors such as age at disease onset, severity of symptoms, and response to treatments also further contribute to etiology. Therefore, we also ran the model using stochastic simulation.

We first compared the output for MMP and TIMP-1 expression over 48 hours with different cytokine treatments and found that although the mean behavior was similar to that of the deterministic model, there was large variability in the expression of MMP-1 mRNA when both IL-1 and OSM were added (see Supplementary Figure 5, available on the *Arthritis & Rheumatology* web site at http://onlinelibrary.wiley.com/doi/10.1002/art.38297/abstract). The level of MMP-1 mRNA was plotted for 50 individual runs, and as can be seen in Supplementary Figure 5D, there was considerable variation in the amount of induction and in the timing of maximal induction. We also ran the model with IL-1 plus OSM with the addition of MMP activator for a simulated time of 14 days. The model predicted a lot of variability in the levels of active MMPs and, hence, variation also in the percentage of collagen released by day 14 (see Supplementary Figure 6). The mean values of 200 stochastic simulations were fairly similar to the deterministic output, although slightly higher values of active MMPs and collagen release were obtained with the stochastic model.

To examine the role of stochastic effects in possible treatments, we carried out stochastic simulation for the TIMP-1 and TIMP-3 overexpression intervention. The model predicted that TIMP-1 overexpression (×10^3^ or more) may significantly delay collagen release but that there would be only a small reduction in the amount of collagen release by day 14 (see Supplementary Figure 7A, available on the *Arthritis & Rheumatology* web site at http://onlinelibrary.wiley.com/doi/10.1002/art.38297/abstract). Conversely, TIMP-3 overexpression (×10^2^ or more) both significantly delayed and reduced the amount of collagen release (see Supplementary Figure 7B). These results are consistent with the deterministic model and could imply that stochastic effects are not important. However, as in the case of no treatments, there was a lot of individual variation in collagen release (as evidenced experimentally [see refs.^3^–^5^]), which could be seen when examining individual simulations (see Supplementary Figure 8). However, with TIMP-3 overexpression, the variability declined with increasing levels of TIMP-3, suggesting that collagen release was consistently reduced by this intervention.

## DISCUSSION

We developed a mathematical model of some of the pathways involved in cartilage degradation based on experimental data from human chondrocytes stimulated with the cytokines IL-1 and/or OSM. The model included sufficient components to explain the synergistic effects of IL-1 and OSM on MMP expression and the antagonistic effects of IL-1 and OSM on TIMP-1 expression (4). The model was also validated using other data on components of the signaling pathways that are transiently activated in response to cytokines. Since arthritis is characterized by irreversible loss of ECM, we also wanted to include collagen and aggrecan degradation in the model.

The addition of cytokines by themselves is not sufficient for collagen breakdown and the release of collagen fragments, since MMPs are synthesized in an inactive form. Activation of proMMPs is mediated by proteases, and activation has been shown to be a key control point in terms of collagenolysis in arthritic cartilage (6). Therefore, we included the addition of a collagenase-activating protease, which we named MMP activator, in our model. We parameterized the model so that when a collagenase-activating protease is included, ≥10% of collagen is degraded by day 14 after stimulation with IL-1 plus OSM, as seen with human cartilage explants (35). We then used the model to simulate various possible interventions, and we examined the effect of these on collagen release.

Our model predicted that the use of receptor inhibitors may not be beneficial because antagonistic receptors do not totally stop the initial signal and because, following receptor activation, downstream signaling is rapid, leading to transcription of target genes such as ADAMTS and MMPs. This prediction was confirmed by published data showing that IL-1Ra inhibits collagen release in tissue from some patients but not from others (36), which confirms that this may not be a useful approach by itself. OSMR antagonists have only recently been developed and have not yet been tested experimentally in a cartilage breakdown model, so these model predictions will be tested when reagents are available.

Simulated blocking of JAK-1 activity, one of the first kinases in the signaling pathways, was not effective unless 100% inhibition was achieved. With 100% inhibition, there was no phosphorylation of STAT-3, so there was no up-regulation of c-Fos. This means that there were only low levels of ADAMTS, MMPs, and MMP activator, which are insufficient to cause collagen release. However, 100% inhibition is unlikely in the clinical setting, and so this intervention may not be very beneficial if administered alone. The prediction for the effect of JAK-1 inhibition on collagen release is yet to be tested in an experimental setting.

Interestingly, the model predicted that inhibition of p38 or JNK activity would be much more effective, as this decreased the amount of phosphorylated c-Jun and c-Fos, respectively, and so inhibited the formation of AP-1 transcription factor complexes. Inhibiting p38 was predicted to be slightly more effective than inhibiting JNK, as this reduced the formation of the more transcriptionally active AP-1 complex, consisting of c-Fos/c-Jun heterodimers. Previous experimental data (not used in the construction of the model) confirmed that p38 inhibition reduces collagen release in a bovine model of cartilage breakdown (34). In this study, we experimentally explored the effectiveness of JNK inhibition, as predicted by the model, and showed that this was indeed effective. However, the JNK inhibitor we used is not entirely specific, so it is not possible to conclude that these effects were solely due to JNK inhibition, although the results confirm the usefulness of computational models to predict effective interventions.

The model was also used to mimic the overexpression of TIMP-1 or TIMP-3 protein. Although we assumed that TIMP-1 mainly inhibits MMPs, whereas TIMP-3 mainly inhibits ADAMTS-4, the model predicted that TIMP-3 overexpression would have a greater effect on reducing collagen release (Figure 5). This was due to our assumption that aggrecan protects collagen from degradation, and the delay in aggrecan release meant that collagen was not accessible for degradation during the time period when MMP-1 and MMP-13 are most active. Therefore, the model suggested that targeting aggrecan release, especially if the intervention is performed at the appropriate time window, is a promising strategy to investigate further. Our predictions are supported by experimental data showing limited benefit from overexpressing TIMP-1 in a mouse model of arthritis (37). Direct inhibition of MMPs with low molecular weight inhibitors proved to be ineffective in patients, as off-target effects were identified (38).

Although we mainly used deterministic simulations in this study, stochastic effects are an important consideration in biologic systems. Our model predicted that the response to IL-1 plus OSM was variable in terms of the levels of active MMPs and collagen release (see Supplementary Figures 6 and 7, available on the *Arthritis & Rheumatology* web site at http://onlinelibrary.wiley.com/doi/10.1002/art.38297/abstract). Stochastic simulations for TIMP-1 and TIMP-3 overexpression generated average behaviors that were similar to those in the deterministic model. TIMP-3 overexpression was much more effective, significantly delaying and reducing collagen release. Although individual simulation results exhibited considerable variability, this was reduced with increasing amounts of TIMP-3 overexpression, which suggests that this treatment is effective at reducing collagen release.

Our model represents a substantial contribution to the development of a systems approach to ECM breakdown, using cartilage as a reference tissue. This tissue is ideal for modeling studies, as it contains a single cell type. The current model is comprehensive, but we used a modeling approach that is very amenable to adding further details and making modifications as subsequent experimental data and new hypotheses emerge. For example, we are aware that there is cross-talk between signaling pathways, that other cytokines can initiate cartilage breakdown, and that other pathways or levels of control (e.g., the role of noncoding RNAs such as microRNAs and their effect on mRNA stability) are implicated, none of which were included in the model. As new experimental data become available, our model can be extended and refined. However, the guiding principle for building models is to capture the essential details without burdening the model with nonessential details (17). For example, we modeled protein synthesis of some proteins (DUSP-1, MMP activator, MKP-1, PP-4, PTPRT, and Sp-1) as one step, omitting details of transcription, where we did not have data concerning mRNA levels.

The predictions generated by the present model are interesting in that intervention at the level of the receptors had little effect. This is supported by the fact that treatment of rheumatoid arthritis patients with IL-1Ra showed only modest beneficial effects (39). Increasing the level of TIMP-1 was equally ineffective, which confirms the data generated when direct inhibition with MMP inhibitors proved to be ineffective in patients and affected other tissues of the joint as well (38). The results with TIMP-3 could suggest that inhibition of the ADAMTS family could be effective in patients. Although this treatment would target aggrecan-degrading enzymes, a reduction in aggrecan release also helps to prevent irreversible collagen release, since collagen is inaccessible to MMPs when protected by aggrecan. Interventions that prevent the transcription of collagenases, particularly by interfering with JNK signaling pathways, had a much greater effect, and we have validated this prediction experimentally, confirming that this pathway may represent tractable therapeutic targets (40).

In conclusion, there is a great need to increase our understanding of the molecular mechanisms involved in cartilage release and to develop new interventions (19). We have shown that computer modeling is an ideal tool to assist in these processes, and there is great potential for future developments of this approach.
