# Supplementary material for: A Computer Simulation Approach to Assessing Therapeutic Intervention Points for the Prevention of Cytokine-Induced Cartilage Breakdown
Source: Arthritis Rheumatol. 2014 Mar 28;66(4):979–89. doi: 10.1002/art.38297 (PMC4033570; doi:10.1002/art.38297)
Supplement: Supplementary file 1 [file art0066-0979-sd1.pdf]

A computer simulation approach for assessing therapeutic intervention points to prevent cytokine-induced cartilage breakdown

C. J. Proctor[1,2], C. Macdonald[3], J. M. Milner[3], A. D. Rowan[1,3], and T. E. Cawston[1,3]

[1]MRC-Arthritis Research UK Centre for Integrated Research into Musculoskeletal Ageing

[2]Institute for Ageing and Health, Newcastle University, Newcastle upon Tyne, UK

[3]Institute of Cellular Medicine, Newcastle University, Newcastle upon Tyne, UK

Supplementary figures:

Figure S1 Network diagram of the IL-1 pathway ……………………………………………………………………………2

Figure S2 Network diagram of the OSM pathway …………………………………………………………………………..3

Figure S3 Network diagram of MMP activation and aggrecan/collagen degradation ………………………4

Figure S4 The kinetics of cFos and cJun induction after IL-1 + OSM stimulation ………………………………5

Figure S5 Stochastic simulations showing effect of IL-1 and/or OSM on MMP and TIMP-1 expression ……6

Figure S6 Stochastic simulations showing effect of MMPActivator on activation of MMPs and collagen release ……7

Figure S7 Stochastic simulations for TIMP overexpression interventions …………………………………………8

Figure S8 Individual stochastic simulations for TIMP overexpression interventions …………………………9

Figure S9 Simulation results used for model validation ……………………………………………………………… 10

Supplementary Tables:

Table S1 List of all the model species ……………………………………………………………………………………. 11

Table S2 List of reactions with kinetic laws for stochastic model ………………………………………………… 13

Table S3 List of parameters ……………………………………………………………………………………………………… 16

Table S4 Simulated treatments ………………………………………………………………………………………………… 17

Table S5 Simulated interventions ……………………………………………………………………………………………… 17

Table S6 Effect of IL1 and OSM antagonist on collagen and aggrecan release after induction by IL-1 + OSM + MMP activator ………………………………………………………………………………………………… 17

Table S7 Experimental data for model construction ………………………………………………………………….. 18

Table S7 Experimental data for model validation ……………………………………………………………………… 18

Figure S1 Network diagram of the IL-1 pathway. IL-1 binds to its receptor and then recruits IRAK2. IRAK2 then binds to TRAF6 which leads to phosphorylation of p38 and JNK. JNK_P phosphorylates cJun which can then form dimers. cJun dimers upregulate MMPs, ADAMTS4, cJun and phosphatases (DUSP16, MKP1 and PP4). DUSP16 and MKP1 dephosphorylate JNK and p38 respectively, and PP4 binds to TRAF6 to inhibits its activity, resulting in inhibition of IL-1 signalling.

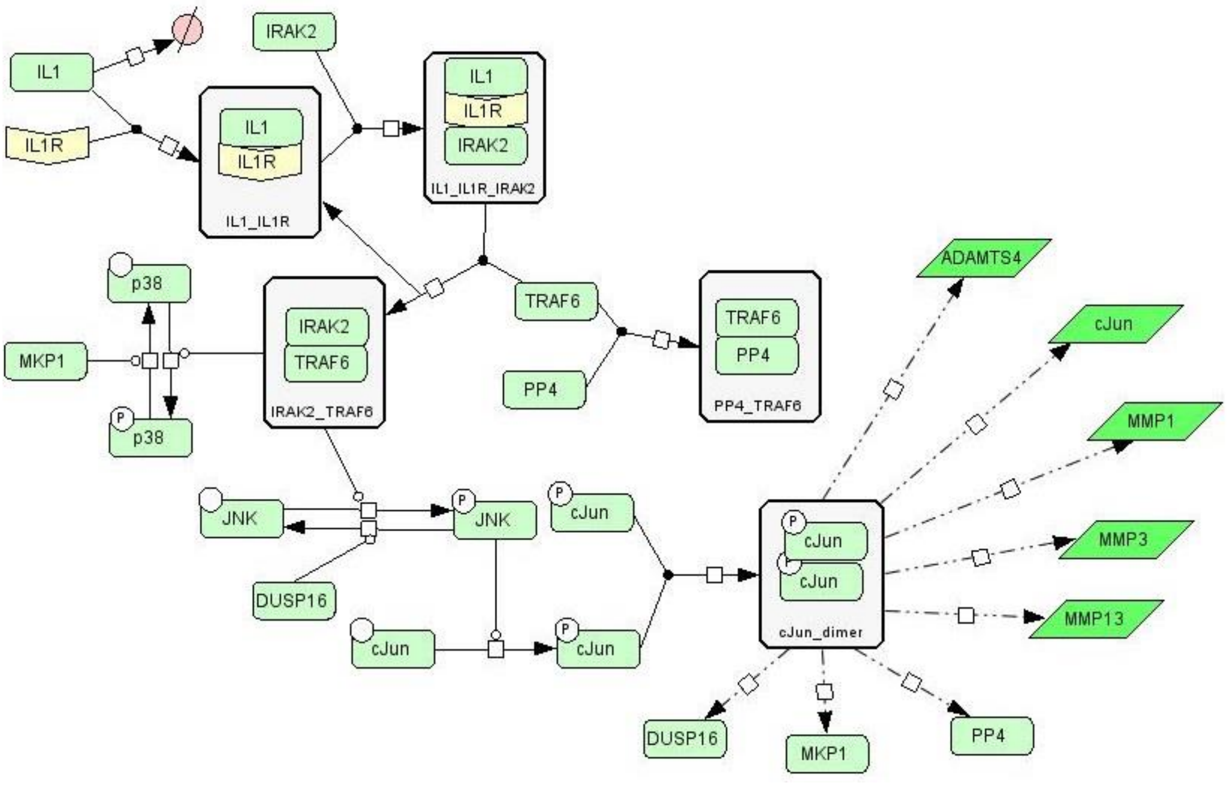

Key:

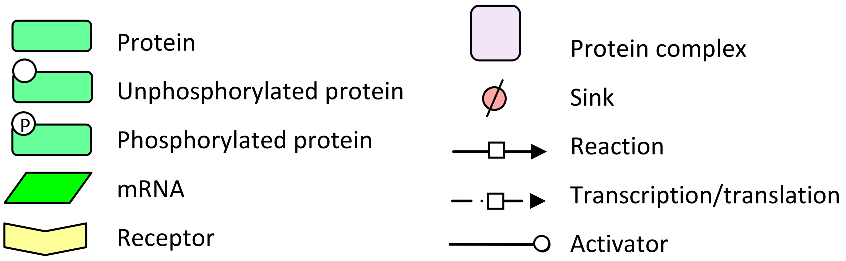

Figure S2 Network diagram of the OSM pathway. OSM binds to its receptor (OSMR) which then phosphorylates JAK1. JAK1 phosphorylates STAT3 which then translocates to the nucleus and upregulates cFos, a phosphatase (PTPRT) and SOCS3. PTPRT inactivates JAK1 and STAT3 and SOCS3 binds to OSMR to inhibit OSM signalling. cFos is phosphorylated by p38 and it can bind to phosphorylated cJun to form AP1 complex (shown as cFos_cJun). This leads to upregulation of MMPs, ADAMTS4, TIMPs, cFos, cJun, phosphatases, a generic MMP activator and the transcription factor, SP1. SP1 binds to TIMP1 promoter to inhibit its transcription (not shown).

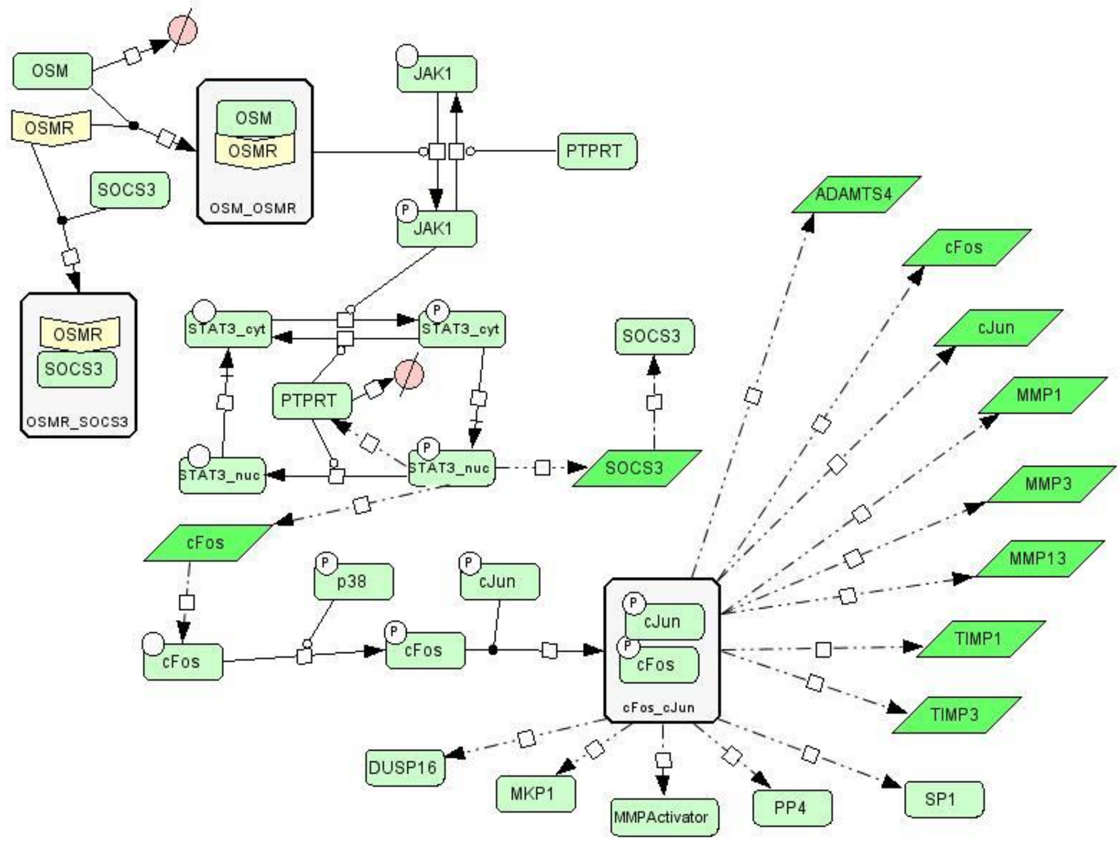

Key:

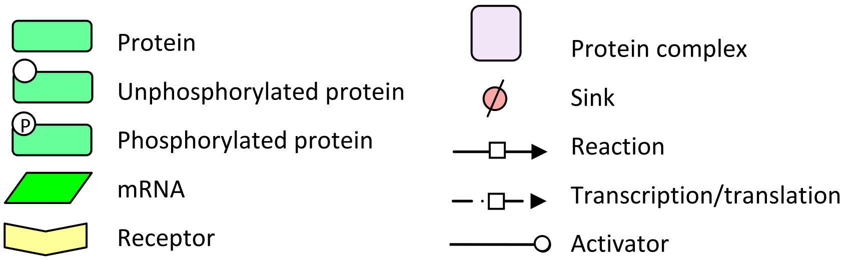

Figure S3 Network diagram of MMP activation and aggrecan/collagen degradation. MMP mRNA is translated into inactive forms of MMPs (proMMP1, proMMP3 and proMMP13). MMPActivator cleaves proMMP1 and proMMP3 to form active MMP1 and MMP3 respectively. MMP3 cleaves proMMP13 to form active MMP13 and also cleaves proMMP1. Collagen is surrounded by aggrecan which protects it from degradation. This is represented in the model by the complex Aggrecan_Collagen. Aggrecan can be released from the complex by ADAMTS4 which results in an aggrecan fragment (AggFrag) and leaves collagen unprotected. Unbound collagen is degraded by MMP1 or MMP13 to produce collagen fragments (ColFrag).

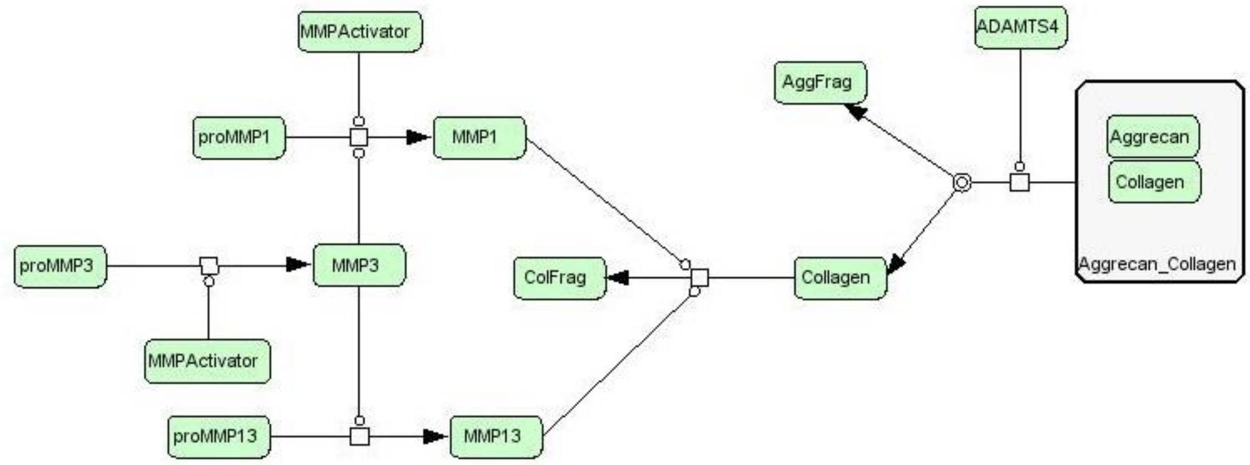

Key:

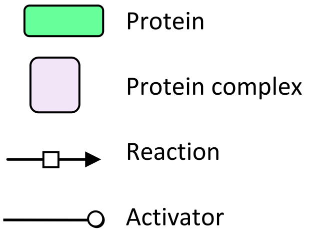

Figure S4 The kinetics of cFos and cJun induction after IL-1 + OSM stimulation.
Chondrocytes were stimulated with IL-1 (0.05 ng/ml) in combination with OSM (10 ng/ml) for the indicated durations. (A) Total RNA was isolated, reverse transcribed and subjected to real-time RT-PCR as described in Litherland *et al*. J Biol Chem 2008; 283:14221-14229. Data are expressed relative to 18S rRNA and presented as fold increase compared to basal expression (mean ± SD, n = 4), where ***, p<0.001; **, p<0.01; *, p<0.05; IL-1+OSM-treated compared to control; ANOVA. PCR data are representative of at least three separate chondrocyte populations.

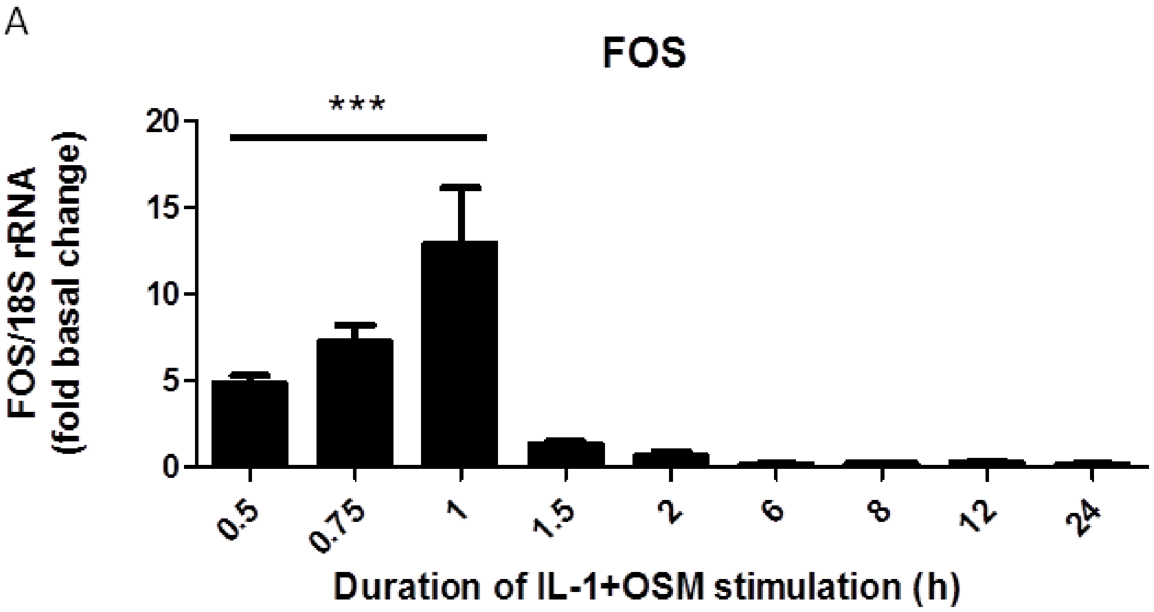

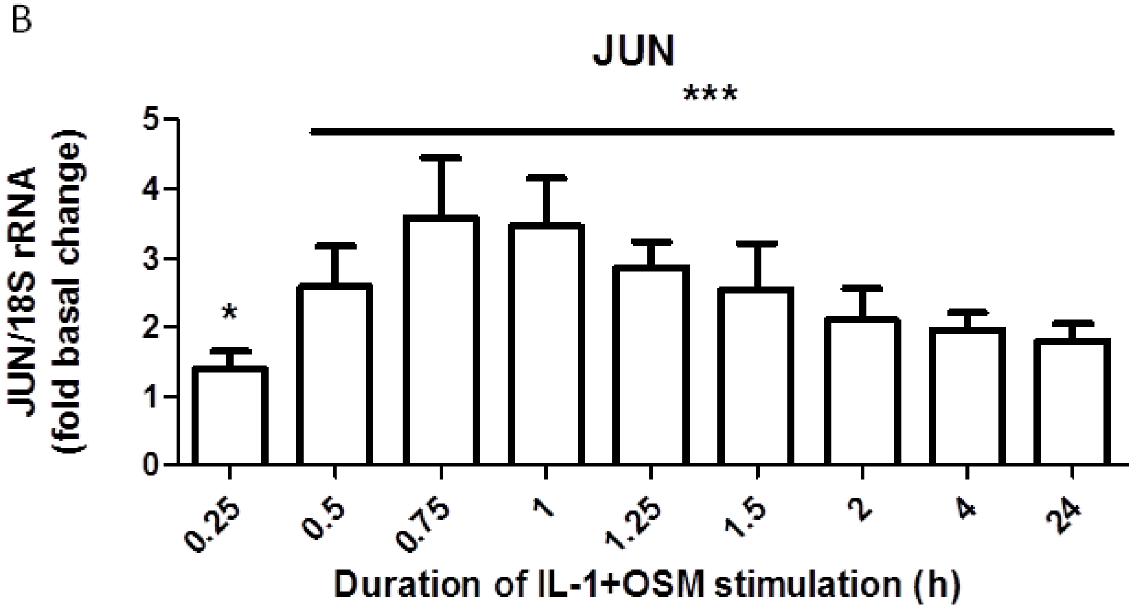

Figure S5 Stochastic simulations showing effect of IL-1 and/or OSM on MMP and TIMP-1 expression. A) IL-1 only, B) OSM only, C) IL-1 + OSM, D) IL-1 + OSM showing MMP-1 mRNA from 50 individual runs Key: blue-MMP-1 mRNA, orange-MMP-13 mRNA, red-TIMP-1 mRNA, A-C: solid curves show mean of 100 simulations, vertical bars indicate ±1 s.d. from the mean.

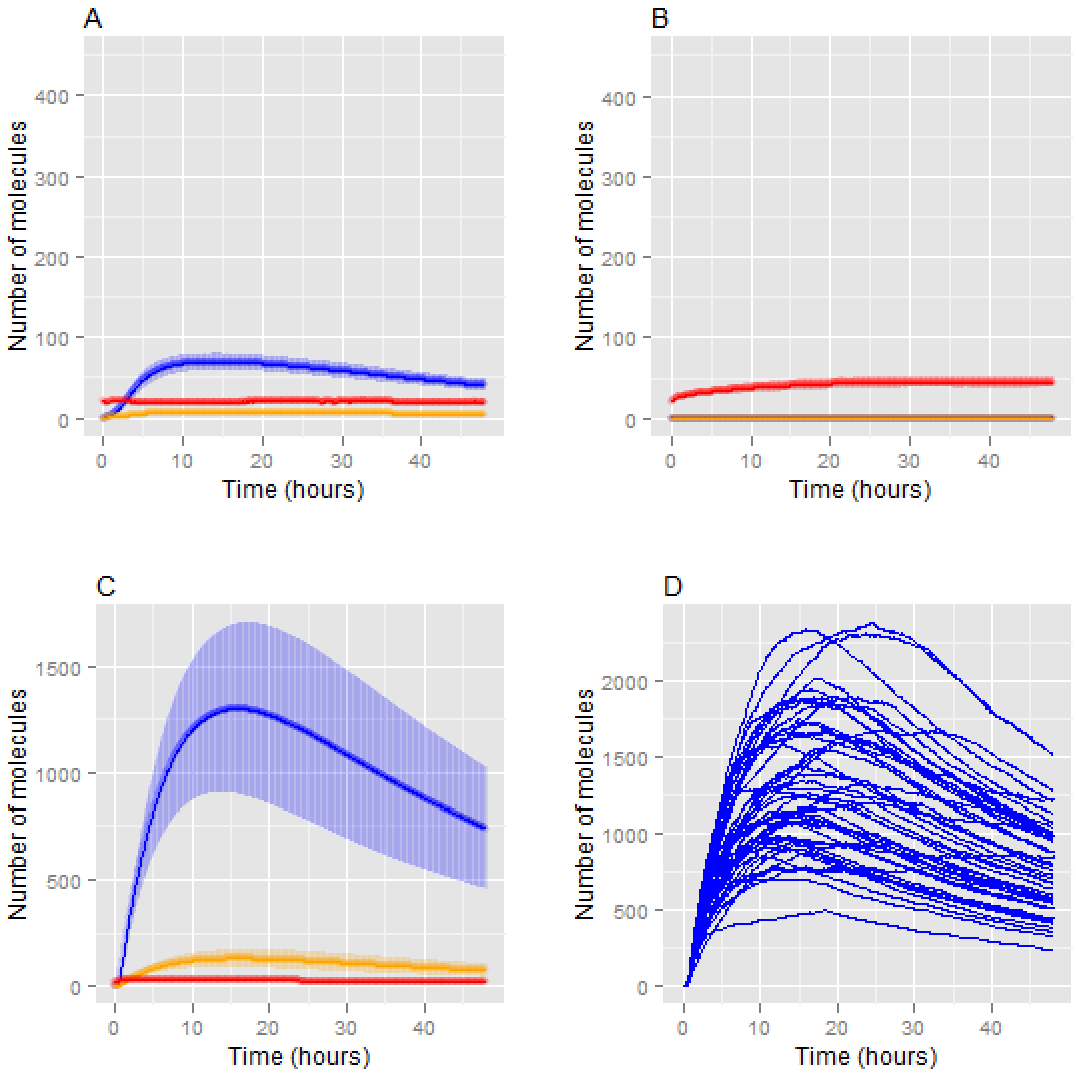

Figure S6 Stochastic simulations showing effect of MMPActivator on activation of MMPs and collagen release. 200 simulations were run over a 14 day period (virtual time). Dark curves show means of 200 simulations, lighter curves are results for 50 individual runs. A) Active MMP-1, B) Active MMP-13, C) Percentage of collagen released.

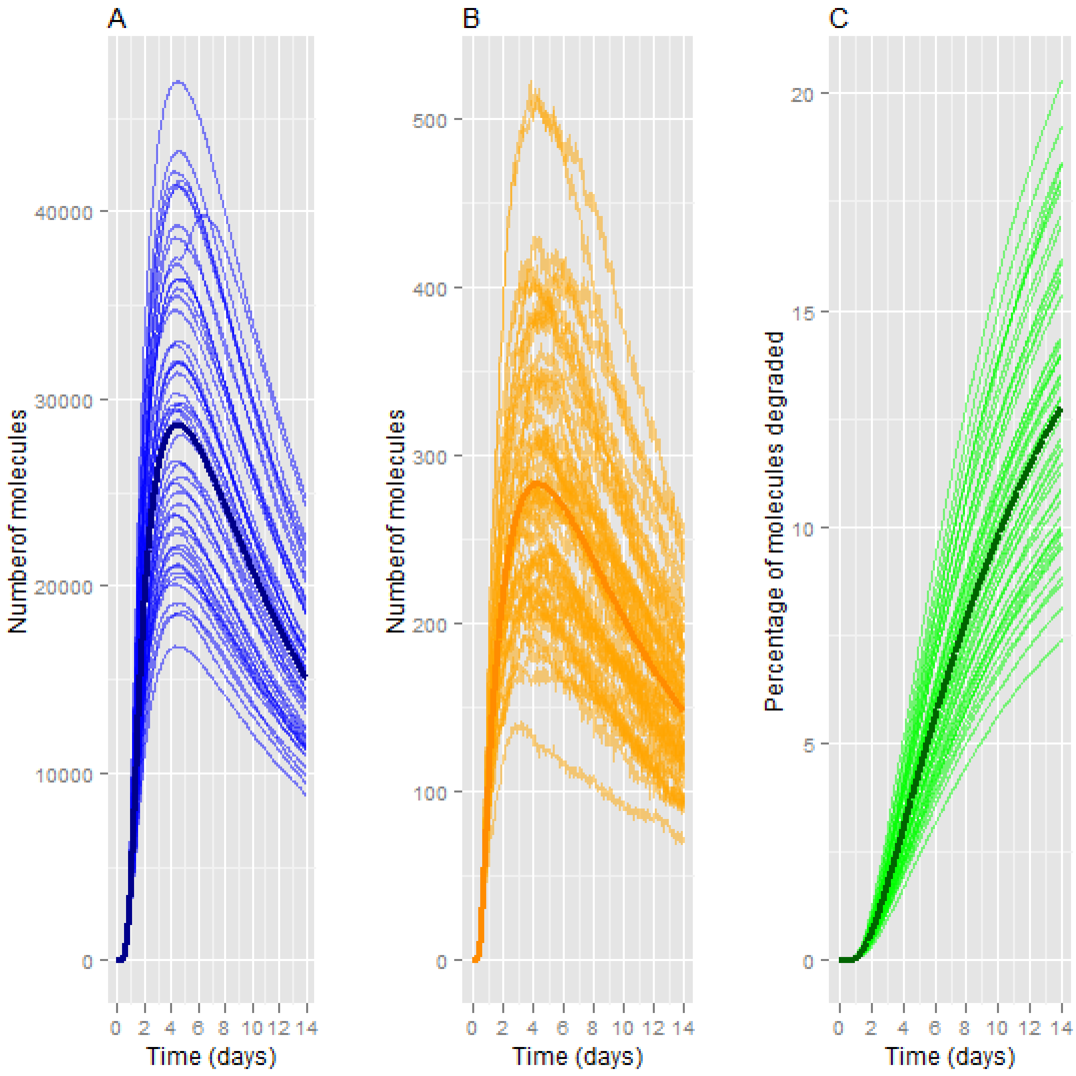

Figure S7 Stochastic simulations for TIMP overexpression interventions. A) TIMP-1 overexpression, B) TIMP-3 overexpression. Plots show the mean of the percentage of collagen released (dark lines) and 95% confidence interval for the mean (light lines) from 200 simulations. Green – basal levels, orange - x10, red - x10^2, blue - x10^3.

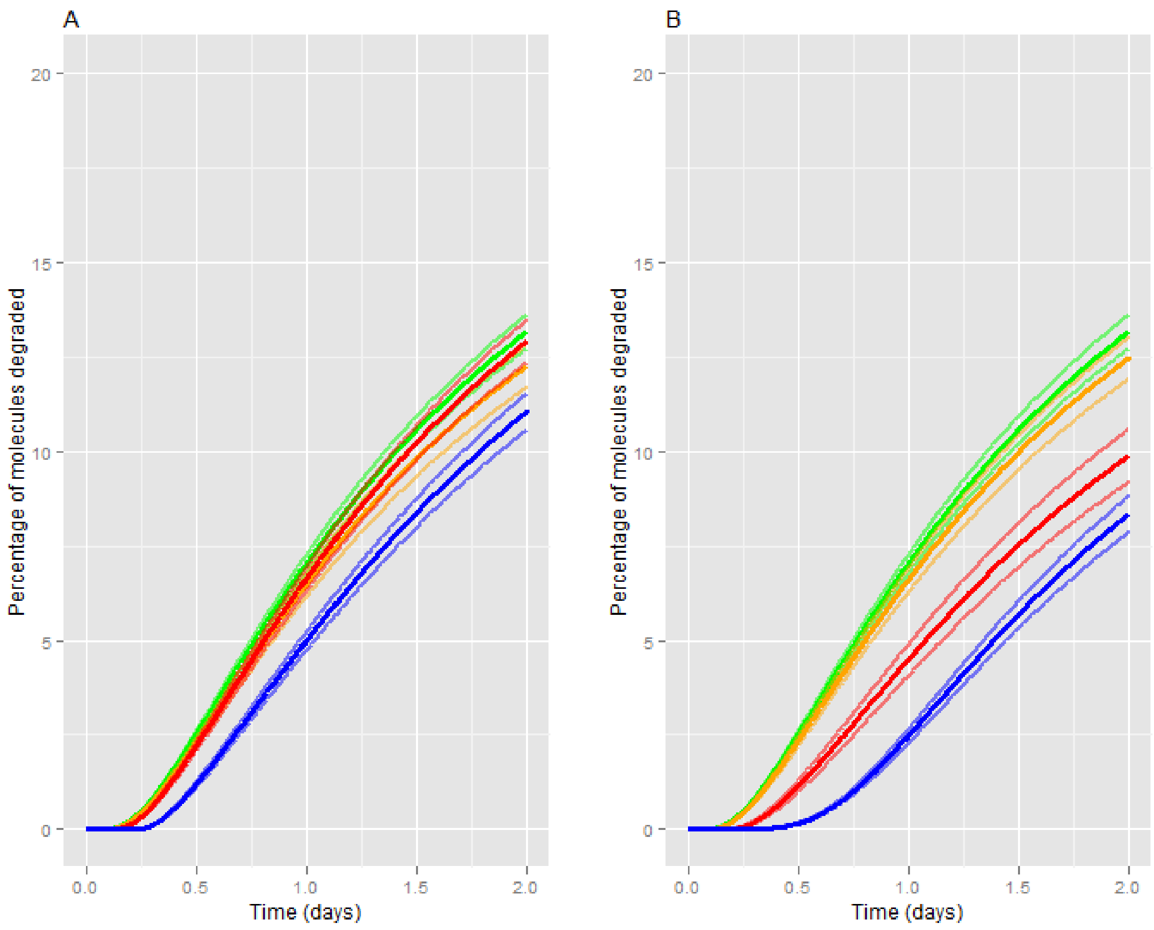

Figure S8 Individual stochastic simulations for TIMP overexpression interventions. Graphs show the percentage of collage released in 50 individual runs out of 200 simulations. A) Basal TIMP-1, B) TIMP-1 x 10, C) TIMP-1 x 10^2, D) TIMP-1 x 10^3, E) Basal TIMP-3, F) TIMP-3 x 10, G) TIMP-3 x 10^2, H) TIMP-3 x 10^3. Dark line in each plot shows the mean from 200 simulations.

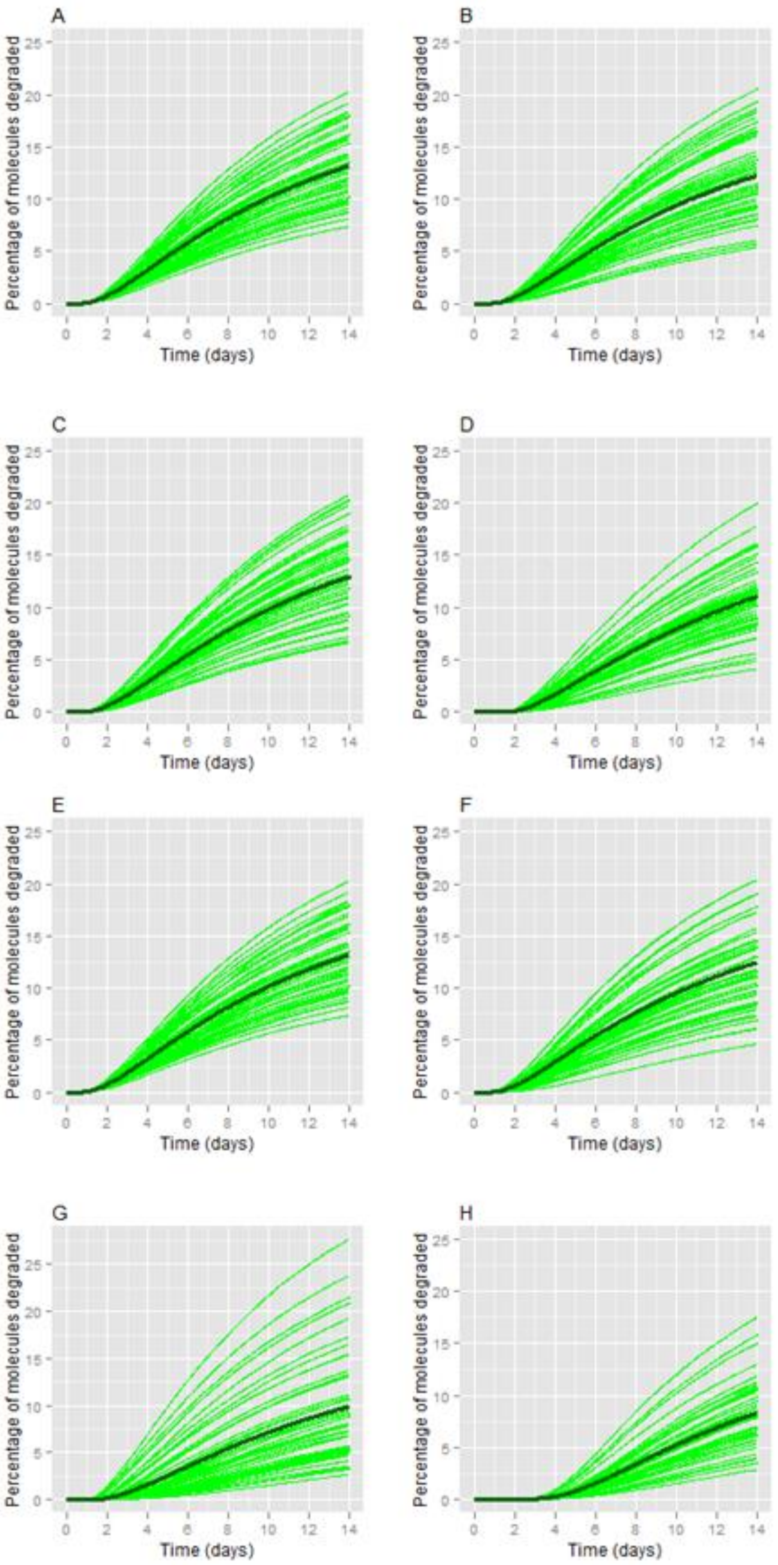

Figure S9 Simulation results for model validation. A) Model with addition of IL-1 only validated against data for levels of phospho-JNK (JNK_P, red curve) and phospho-cJun (cJun_P+cJun_dimers, blue curve. As in the experimental data (Table S7), phospho-JNK is rapidly induced and peaks at about 30 minutes and returns to basal by 6 hours, whereas phospho-cJun peaks later and remains above basal at 6 hours. B) Model with addition of IL-1 + OSM validated against experimental data for phospho-STAT (STAT3_P_cyt+STAT3_P_nuc, black curve), phospho-JAK1 (magenta curve) and phospho-p38 (green curve). As in the experimental data (Table S7), JAK1 is rapidly induced, peaks at about 15 minutes and returns to basal by 1 hour. STAT3 is also rapidly induced, peaking early but is inactivated more slowly than the other kinases. Phospho-p38 peaks slightly later than in the experimental data (at 30 minutes rather than 15 minutes) but this is not surprising as we have omitted many of the pathways leading to activation of p38 in this model. Inactivation of p38 takes longer than 1 hour which is in agreement with the experimental data used for validation (see Table S7 for references).

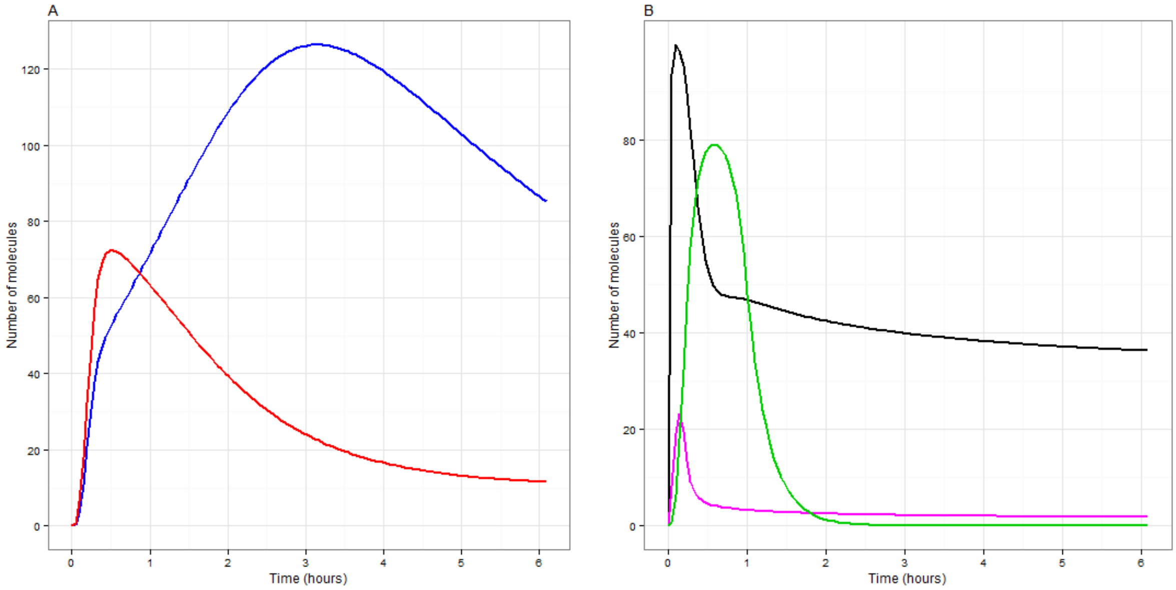

Table S1 List of all the model species

Species name	Description	Database term	Initial value
ADAMTS4	A disintegrin and metalloproteinase with thrombospondin motifs 4. Enzyme that cleaves aggrecan.	O75173	0
ADAMTS4_mRNA	mRNA of ADAMTS-4.	O75173	0
ADAMTS4_TIMP1	TIMP-1 bound to ADAMTS-4.	O75173, P01033	0
ADAMTS4_TIMP3	TIMP-3 bound to ADAMTS-4.	O75173, P35625	0
Aggrecan	Proteoglycan, component of the extracellular matrix (this is bound to collagen in model).	P16112	-
AggFrag	Species to represent aggrecan fragments	-	0
Aggrecan_collagen2	Complex to represent protection of collagen 2 by aggrecan	P16112, P02458	100000
cFos	Nuclear phosphoprotein	P01100	0
cFos_cJun	cFos bound to cJun to form AP1 transcription factor complex	P01100, P05412	0
cFos_mRNA	mRNA of cFos	P01100	0
cFos_P	Phosphorylated cFos	P01100	0
cJun	Transcription factor (inactive form)	P05412	100
cJun_dimer	Dimer of cJun_P (active form)	P05412	0
cJun_mRNA	mRNA of cJun	P05412	5
cJun_P	Phosphorylated cJun	P05412	0
Collagen2	Collagen 2, component of the extracellular matrix. This is unprotected pool.	P02458	0
ColFrag	Species to represent collagen fragment	-	0
DUSP16	Phosphatase	Q9BY84	0
IL1	Cytokine – interleukin-1α	P01583	0
IL1R	Interleukin-1 receptor 1 (IL-1R1)	P14778	100
IL1_IL1R	IL-1α bound to its receptor	P01583, P14778	0
IL1_IL1R_IRAK2	IL-1/IL-1R complex bound by IRAK2	P01583, P14778, O43187	0
IL1Ra	IL-1R antagonist (IL1RN)	P18510	0
IL1_IL1Ra	IL-1α bound to antagonist receptor	P01583, P18510	0
IRAK2	Interleukin-1 receptor associated kinase 2	O43187	100
IRAK2_TRAF6	IRAK2 bound toTRAF6	O43187, Q9Y4K3	0
IRAK2_TRAF6_PP4	IRAK2/TRAF6 complex bound by PP4	O43187, Q9Y4K3, P60510	0
JAK1	Tyrosine-protein kinase JAK1	P23458	100
JAK1_P	Phosphorylated JAK1	P23458	0
JNK	c-Jun N-terminal kinase 1 (JNK1, MAPK8)	P45983	100
JNK_P	Phosphorylated JNK	P45983	0
MMPActivator	Generic collagenase activator		0
MKP1	Mitogen-activated kinase phosphatase 1, Dual specificity protein 1 (*DUSP1*)	P28562	0
MMP1	Matrix metalloproteinase-1	P03956	0
MMP1_mRNA	mRNA of MMP-1	P03956	0

MMP1_TIMP1	TIMP-1 bound to MMP-1	P03956, P01033	0
MMP1_TIMP3	TIMP-3 bound to MMP-1	P03956, P35625	0
MMP3	Matrix metalloproteinase-3	P08254	0
MMP3_mRNA	mRNA of MMP-3	P08254	0
MMP3_TIMP1	TIMP-1 bound to MMP-3	P08254, P01033	0
MMP3_TIMP3	TIMP-3 bound to MMP-3	P08254, P35625	0
MMP13	Matrix metalloproteinase-13	P45452	0
MMP13_mRNA	mRNA of MMP-13	P45452	0
MMP13_TIMP1	TIMP-1 bound to MMP-13	P45452, P01033	0
MMP13_TIMP3	TIMP-3 bound to MMP-13	P45452, P35625	0
OSM	Oncostatin M	P13725	0
OSMR	OSM receptor	Q99650	100
OSM_OSMR	OSM bound to its receptor	P13725, Q99650	0
OSMRa	OSM receptor antagonist	-	0
OSM_OSMRa	OSM bound to OSMRa	P13725	0
OSMR_SOCS3	OSM receptor bound by SOCS3	Q99650, O14543	0
P38	P38 MAPK kinase (MAPK14)	Q16539	100
P38_P	Phosphorylated p38	Q16539	0
PP4	Serine/theorine-protein phosphatase 4	P60510	0
proMMP1	Inactive form of MMP-1	P03956	0
proMMP3	Inactive form of MMP-3	P08254	0
proMMP13	Inactive form of MMP-13	P45452	0
PTPRT	Receptor-type tyrosine-protein phosphatase T	O14522	0
SOCS3	Suppressor of cytokine signalling 3	O14543	0
SOCS3_mRNA	mRNA of SOCS3	O14543	0
SP1	Transcription factor SP1	P08047	0
SP1_TIMP1_DNA	SP1 bound to repressive element of TIMP-1 promoter	P08047, P01033	0
STAT3_cyt	Cytoplamic pool of Signal transducer and activator of transcription 3 (STAT3)	P40763	100
STAT3_nuc	Nuclear pool of STAT3	P40763	0
STAT3_P_cyt	Cytoplasmic pool of phosphorylated STAT3	P40763	0
STAT3_P_nuc	Nuclear pool of phosphorylated STAT3	P40763	0
TIMP1	Tissue inhibitor of metalloproteinases 1	P01033	200
TIMP1_DNA	SP1 binding site of TIMP-1 DNA	-	2
TIMP1_mRNA	mRNA of TIMP-1	P01033	10
TIMP3	Tissue inhibitor of metalloproteinases 3	P35625	200
TIMP3_mRNA	mRNA of TIMP-3	P35625	10
TRAF6	TNF receptor- associated factor 6 (TRAF6)	Q9Y4K3	100
TRAF6_PP4	TRAF6 bound by PP4	Q9Y4K3, P60510	

Table S2 List of reactions with kinetic laws for stochastic model. Rate laws are identical for deterministic model except for cJun dimerisation reaction.

Reaction name	Reactants	Products	Kinetic law
IL1binding	IL1, IL1R	IL1_IL1R	$k_{binIL1IL1R}$*IL1*IL1R
IL1release	IL1_IL1R	IL1, IL1R	$k_{relIL1IL1R}$*IL1_IL1R
IL1degradation	IL1	Sink	k_{degIL1}*IL1
IRAK2binding	IL1_IL1R, IRAK2	IL1_IL1R_IRAK2	$k_{binIRAK2}$*IL1_IL1R*IRAK2
IRAK2release	IL1_IL1R_IRAK2	IL1_IL1R, IRAK2	$k_{relIRAK2}$*IL1_IL1R_IRAK2
TRAF6binding[a]	IL1_IL1R_IRAK2, TRAF6	IL1_IL1R, IRAK2_TRAF6	$k_{binTRAF6}$*IL1_IL1R_IRAK * TRAF6
TRAF6 inhibition via PP4 binding[a]	PP4, TRAF6	PP4_TRAF6	$k_{inhibTRAF6}$*PP4*TRAF6
IRAK2_TRAF6 inhibition[a]	IRAK2_TRAF6, PP4	IRAK2_TRAF6_PP4	$k_{inhibTRAF6}$*IRAK2_TRAF6 *PP4
JNKphosphorylation	IRAK2_TRAF6, JNK	IRAK2_TRAF6, JNK_P	$k_{phosJNK}$*IRAK2_TRAF6 *JNK
JNK dephosphorylation	JNK_P	JNK	$k_{dephosJNK}$*JNK_P
JNKdephosphorylation ByDUSP16	DUSP16, JNK_P	DUSP16, JNK	$k_{dephosJNKDUSP16}$*JNK_P *DUSP16
cJun phosphorylation	cJun, JNK_P	cJun_P, JNK_P	$k_{phoscJun}$*cJun*JNK_P
cJun dephosphorylation	cJun_P	cJun	$k_{dephoscJun}$*cJun_P
cJun dimerization	2 cJun_P	cJun_dimer	$k_{dimercJun}$*cJun_P *(cJun_P-1)*0.5[c]
cJun dedimerization	cJun_dimer	2 cJun_P	$k_{dedimercJun}$*cJun_dimer
cJun basal transcription	Source	cJun_mRNA	$k_{synbasalcJunmRNA}$*Source
cJun transcription via cJun dimers	cJun_dimer	cJun_dimer, cJun_mRNA	$k_{syncJunmRNAcJun}$*cJun_dimer
cJun transcription via AP1	cFos_cJun	cFos_cJun, cJun_mRNA	$k_{syncJunmRNA}$* cFos_cJun
cJun mRNA degradation	cJun_mRNA	Sink	$k_{degcJunmRNA}$*cJun_mRNA
cJun translation	cJun_mRNA	cJun_mRNA, cJun	$k_{syncJun}$*cJun_mRNA
cJun degradation	CJun	Sink	$k_{degcJun}$*cJun
p38 phosphorylation	IRAK2_TRAF6, p38	IRAK2_TRAF6, p38_P	$k_{phosp38}$*IRAK2_TRAF6 *p38
p38 dephosphorylation	p38_P	p38	$k_{dephosp38}$*p38_P
p38 dephosphorylation by MKP1	MKP1, p38_P	MKP1, p38	$k_{dephosp38MKP1}$*MKP1* p38_P
OSM/OSMR binding[a]	OSM, OSMR	OSM_OSMR	$k_{binOSMOSMR}$*OSM*OSMR
OSM degradation	OSM	Sink	k_{degOSM}*OSM
JAK1 activation	JAK1, OSM_OSMR	JAK1_P, OSM_OSMR	$k_{phosJAK1}$*JAK1 *OSM_OSMR
JAK1 inactivation	JAK1_P	JAK1	$k_{dephosJAK1}$*JAK1_P
JAK1 inactivation by PTPRT	JAK1_P, PTPRT	JAK1, PTPRT	$k_{dephosJAK1PTPRT}$*JAK1_P *PTPRT
STAT3 phosphorylation in cytosol	JAK1_P, STAT3_cyt	JAK1_P, STAT3_P_cyt	$k_{phosSTAT3}$*JAK1_P *STAT3_cyt
STAT3_P_cyt dephosphorylation	STAT3_P_cyt	STAT3_cyt	$k_{dephosSTAT3}$*STAT3_P_cyt
STAT3_cyt dephosphorylation by PTPRT	PTPRT, STAT3_P_cyt	PTPRT, STAT3_cyt	$k_{dephosSTAT3PTPRT}$*PTPRT *STAT3_P_cyt
STAT3 transport to nucleus	STAT3_P_cyt	STAT3_P_nuc	$k_{cyt2nucSTAT3}$*STAT3_P_cyt
STAT3_P_nuc dephosphorylation	STAT3_P_nuc	STAT3_nuc	$k_{dephosSTAT3nuc}$ *STAT3_P_nuc
STAT3_P_nuc dephosphorylation by PTPRT	PTPRT, STAT3_P_nuc	PTPRT,STAT3_nuc	$k_{dephosSTAT3nucPTPRT}$ *PTPRT*STAT3_P_nuc

STAT3 transport from nucleus	STAT3_nuc	STAT3_cyt	$k_{nuc2cytSTAT3}$ *STAT3_P_nuc
cFos transcription via STAT3	STAT3_P_nuc	cFos_mRNA, STAT3_P_nuc	$k_{syncFosmRNASTAT3}$ *STAT3_P_nuc
cFos transcription via AP1	cFos_cJun	cFos_cJun, cFos_mRNA	$k_{syncFosmRNA}$ *cFos_cJun
cFos mRNA degradation	cFos_mRNA	Sink	$k_{degcFosmRNA}$ *cFos_mRNA
cFos translation	cFos_mRNA	cFos, cFos_mRNA	$k_{syncFos}$ *cFos_mRNA
cFos degradation	CFos	Sink	$k_{degcFos}$ *cFos
cFos phosphorylation via p38	cFos, p38_P	cFos_P, p38_P	$k_{phoscFos}$ *cFos*p38_P
cFos dephosphorylation	cFos_P	cFos	$k_{dephoscFos}$ *cFos_P
cFos dephosphorylation by DUSP16	cFos_P, DUSP16	cFos, DUSP16	$k_{dephoscFosDUSP16}$ *cFos_P * DUSP16
cFos/cJun binding[a]	cFos_P, cJun_P	cFos_cJun	$k_{bincFoscJun}$ * cFos_P * cJun_P
ADAMTS4 transcription via cJun dimers	cJun_dimer	ADAMTS4_mRNA, cJun_dimer	$k_{synADAMTS4mRNAcJun}$ * cJun_dimer
ADAMTS4 transcription via AP-1	cFos_cJun	ADAMTS4_mRNA, cFos_cJun	$k_{synADAMTS4mRNA}$ * cFos_cJun
ADAMTS4 translation	ADAMTS4_mRNA	ADAMTS4_mRNA, ADAMTS4	$k_{synADAMTS4}$ * ADAMTS4_mRNA
ADAMTS4 mRNA degradation	ADAMTS4_mRNA	Sink	$k_{degADAMTS4mRNA}$ * ADAMTS4_mRNA
ADAMTS4 degradation	ADAMTS4	Sink	$k_{degADAMTS4}$ * ADAMTS4
DUSP16 synthesis via AP-1	cFos_cJun	cFos_cJun , DUSP16	$k_{synDUSP16}$ * cFos_cJun
DUSP16 synthesis via cJun dimers	cJun_dimer	cJun_dimer, DUSP16	$k_{synDUSP16cJun}$ *cJun_dimer
DUSP16 degradation	DUSP16	Sink	$k_{degDUSP16}$ *DUSP16
MKP1 synthesis via AP-1	cFos_cJun	cFos_cJun, MKP1	$k_{synMKP1}$ *cFos_cJun
MKP1 synthesis via cJun dimers	cJun_dimer	cJun_dimer, MKP1	$k_{synMKP1cJun}$ *cJun_dimer
MKP1 degradation	MKP1	Sink	$k_{degMKP1}$ *MKP1
PP4 synthesis via AP-1	cFos_cJun	cFos_cJun, PP4	k_{synPP4} *cFos_cJun
PP4 synthesis via cJun dimers	cJun_dimer	cJun_dimer, PP4	$k_{synPP4cJun}$ *cJun_dimer
PP4 degradation	PP4	Sink	k_{degPP4} *PP4
PTPRT synthesis via STAT3	STAT3_P_nuc	PTPRT, STAT3_P_nuc	$k_{synPTPRT}$ *STAT3_P_nuc
PTPRT degradation	PTPRT	Sink	$k_{degPTPRT}$ *PTPRT
SOCS3 transcription via STAT3	STAT3_P_nuc	SOCS3_mRNA, STAT3_P_nuc	$k_{synSOCS3mRNA}$ *STAT3_P_nuc
SOCS3 mRNA degradation	SOCS3_mRNA	Sink	$k_{degSOCS3mRNA}$ *SOCS3_mRNA
SOCS3 translation	SOCS3_mRNA	SOCS3, SOCS3_mRNA	$k_{synSOCS3}$ *SOCS3_mRNA
SOCS3 degradation	SOCS3	Sink	$k_{degSOCS3}$ *SOCS3
OSMR/SOCS3 binding[a]	OSMR, SOCS3	OSMR_SOCS3	$k_{binSOCS3OSMR}$ *OSMR*SOCS3
MMP1 transcription via cJun dimers[b]	cJun_dimer	cJun_dimer, MMP1_mRNA	$k_{synMMP1mRNAcJun}$ *cJun_dimer
MMP1 transcription via AP1[b]	cFos_cJun	cFos_cJun, MMP1_mRNA	$k_{synMMP1mRNA}$ *cFos_cJun
MMP1 mRNA degradation[b]	MMP1_mRNA	Sink	$k_{degMMP1mRNA}$ *MMP1_mRNA
MMP1 translation[b]	MMP1_mRNA	proMMP1, MMP1_mRNA	$k_{synMMP1}$ *MMP1_mRNA

MMP1 degradation[b]	MMP1	Sink	$k_{degMMP1}$ *MMP1
proMMP1 cleavage by MMPActivator	MMPActivator, proMMP1	MMPActivator, MMP1	$k_{actMMP1}$ *MMPActivator*proMMP1
proMMP1 cleavage by MMP3	MMP3, proMMP1	MMP3, MMP1	$k_{actMMP1mmp3}$ *MMP3*proMMP1
proMMP3 cleavage by MMPActivator	MMPActivator, proMMP3	MMPActivator, MMP3	$k_{actMMP3}$ *MMPActivator*proMMP3
proMMP13 cleavage by MMP3	MMP3, proMMP13	MMP3, MMP13	$k_{actMMP13mmp3}$ *MMP3*proMMP13
MMPActivator synthesis via AP1	cFos_cJun	cFos_cJun, MMPActivator	$k_{synMMPActivator}$ * cFos_cJun
MMPActivator degradation	MMPActivator	Sink	$k_{degMMPActivator}$ * MMPActivator
SP1 synthesis	cFos_cJun	cFos_cJun, SP1	k_{synSP1} * cFos_cJun
SP1 degradation	SP1	Sink	k_{degSP1} * SP1
TIMP1 basal transcription	TIMP1_DNA	TIMP1_DNA, TIMP1_mRNA	$k_{synbasalTIMP1mRNA}$ *TIMP1_DNA
TIMP1 transcription via STAT3	STAT3_P_nuc, TIMP1_DNA	STAT3_P_nuc, TIMP1_DNA, TIMP1_mRNA	$k_{synTIMP1mRNAStat3}$ * STAT3_P_nuc * TIMP1_DNA
TIMP1 transcription via AP1	cFos_cJun, TIMP1_DNA	cFos_cJun, TIMP1_DNA TIMP1_mRNA	$k_{synTIMP1mRNA}$ * cFos_cJun * TIMP1_DNA
TIMP1 mRNA degradation	TIMP1_mRNA	Sink	$k_{degTIMP1mRNA}$ * TIMP1_mRNA
TIMP1 translation	TIMP1_mRNA	TIMP1, TIMP1_mRNA	$k_{synTIMP1}$ * TIMP1_mRNA
TIMP1 degradation	TIMP1	Sink	$k_{degTIMP1}$ *TIMP1
SP1_TIMP1_DNA binding[a]	SP1, TIMP1_DNA	SP1_TIMP1_DNA	$k_{binSP1TIMP1DNA}$* SP1 * TIMP1_DNA
TIMP3 basal transcription	Source	TIMP3_mRNA	$k_{synbasalTIMP3mRNA}$ * Source
TIMP3 transcription via STAT3	STAT3_P_nuc	STAT3_P_nuc, TIMP3_mRNA	$k_{synTIMP3mRNAStat3}$ * STAT3_P_nuc
TIMP3 transcription via AP1	cFos_cJun	cFos_cJun, TIMP3_mRNA	$k_{synTIMP3mRNA}$ * cFos_cJun
TIMP3 mRNA degradation	TIMP3_mRNA	Sink	$k_{degTIMP3mRNA}$ * TIMP3_mRNA
TIMP3 translation	TIMP3_mRNA	TIMP3, TIMP3_mRNA	$k_{synTIMP3}$ * TIMP3_mRNA
TIMP3 degradation	TIMP3	Sink	$k_{degTIMP3}$ * TIMP3
MMP1 inhibition by TIMP1[a,b]	MMP1, TIMP1	MMP1_TIMP1	$k_{inhibMMP1TIMP1}$ * MMP1* TIMP1
ADAMTS4 inhibition by TIMP1[a]	ADAMTS4, TIMP1	ADAMTS4_TIMP1	$k_{inhibADAMTS4TIMP1}$ * ADAMTS4 * TIMP1
MMP1 inhibition by TIMP3[a,b]	MMP1, TIMP3	MMP1_TIMP3	$k_{inhibMMP1TIMP3}$ * MMP1* TIMP3
ADAMTS4 inhibtion by TIMP3[a]	ADAMTS4, TIMP3	ADAMTS4_TIMP3	$k_{inhibADAMTS4TIMP3}$ * ADAMTS4 * TIMP3
Aggrecan degradation by ADAMTS4	ADAMTS4, Aggrecan_Collagen2	ADAMTS4, AggFrag, Collagen2	$k_{degAggrecan}$ * ADAMTS4* Aggrecan_Collagen2
Collagen degradation by MMP1	Collagen2, MMP1	ColFrag, MMP1	$k_{degCollagen2mmp1}$ * Collagen2 * MMP1
Collagen degradation by MMP13	Collagen2, MMP13	ColFrag, MMP13	$k_{degCollagen2mmp13}$ * Collagen2 * MMP13

[a]Reversible reaction not shown, [b]A similar reaction also occurs for MMP3 and MMP13, [c]rate law for deterministic model is k_{dimer} * [cJun]^2/2.

Table S3 List of parameters

Parameter	Value[a]	Parameter	Value[a]	Parameter	Value[a]
$k_{actMMP13mmp3}$	5.0E-8 mol^{-1}s^{-1}	$k_{dephoscFos}$	1.0E-4 s^{-1}	$k_{relSOCS3OSMR}$	1.0E-5 s^{-1}
$k_{actMMP1}$	1.0E-9 mol^{-1}s^{-1}	$k_{dephoscFosDUSP16}$	1.0E-4 mol^{-1}s^{-1}	$k_{relSP1TIMP1DNA}$	5.0E-6 s^{-1}
$k_{actMMP1mmp3}$	1.0E-8 mol^{-1}s^{-1}	$k_{dephoscJun}$	1.0E-2 s^{-1}	$k_{relTRAF6}$	1.0E-4 s^{-1}
$k_{actMMP3}$	4.0E-6 mol^{-1}s^{-1}	$k_{dephosJAK1}$	4.0E-4 s^{-1}	$k_{relTRAF6PP4}$	1.0E-6 s^{-1}
$k_{bincFoscJun}$	5.0E-5 mol^{-1}s^{-1}	$k_{dephosJAK1PTPRT}$	4.0E-3 mol^{-1}s^{-1}	$k_{synADAMTS4}$	5.0E-4 s^{-1}
$k_{binIL1IL1R}$	1.0E-4 mol^{-1}s^{-1}	$k_{dephosJNK}$	1.0E-3 s^{-1}	$k_{synADAMTS4mRNA}$	5.0E-4 s^{-1}
$k_{binIL1IL1Ra}$	1.0E-4 mol^{-1}s^{-1}	$k_{dephosJNKDUSP16}$	1.0E-3 mol^{-1}s^{-1}	$k_{synADAMTS4mRNAcJun}$	4.0E-6 s^{-1}
$k_{binIRAK2}$	5.0E-5 mol^{-1}s^{-1}	$k_{dephosp38}$	1.0E-3 s^{-1}	$k_{synbasalcJunmRNA}$	1.5E-2 mol s^{-1}
$k_{binOSMOSMR}$	1.0E-5 mol^{-1}s^{-1}	$k_{dephosp38MKP1}$	1.0E-5 mol^{-1}s^{-1}	$k_{synbasalTIMP1mRNA}$	1.4E-4 s^{-1}
$k_{binOSMOSMRa}$	1.0E-4 mol^{-1}s^{-1}	$k_{dephosSTAT3}$	1.0E-5 s^{-1}	$k_{synbasalTIMP3mRNA}$	2.8E-4 mol s^{-1}
$k_{binSOCS3OSMR}$	5.0E-3 mol^{-1}s^{-1}	$k_{dephosSTAT3nuc}$	1.0E-7 s^{-1}	$k_{syncFos}$	1.0E-3 s^{-1}
$k_{binSP1TIMP1DNA}$	1.0E-5 mol^{-1}s^{-1}	$k_{dephosSTAT3nucPTPRT}$	5.0E-4 mol^{-1}s^{-1}	$k_{syncFosmRNA}$	5.0E-6 s^{-1}
$k_{binTRAF6}$	1.0E-5 mol^{-1}s^{-1}	$k_{dephosSTAT3PTPRT}$	8.0E-4 mol^{-1}s^{-1}	$k_{syncFosmRNASTAT3}$	5.0E-2 s^{-1}
$k_{cyt2nucSTAT3}$	1.0E-3 s^{-1}	$k_{dimercJun}$	5.0E-5 mol^{-1}s^{-1}	$k_{syncJun}$	2.6E-3 s^{-1}
$k_{dedimercJun}$	1.0E-2 s^{-1}	$k_{inhibADAMTS4TIMP1}$	3.0E-6 mol^{-1}s^{-1}	$k_{syncJunmRNA}$	1.25E-2 s^{-1}
$k_{degADAMTS4}$	5.0E-5 s^{-1}	$k_{inhibADAMTS4TIMP3}$	5.0E-4 mol^{-1}s^{-1}	$k_{syncJunmRNAcJun}$	5.0E-3 s^{-1}
$k_{degADAMTS4mRNA}$	1.4E-5 s^{-1}	$k_{inhibMMP13TIMP1}$	3.0E-7 mol^{-1}s^{-1}	$k_{synDUSP16}$	5.0E-3 s^{-1}
$k_{degAggrecan}$	2.0E-7 mol^{-1}s^{-1}	$k_{inhibMMP13TIMP1}$	1.0E-8 mol^{-1}s^{-1}	$k_{synDUSP16cJun}$	2.0E-4 s^{-1}
$k_{degcFos}$	2.0E-4 s^{-1}	$k_{inhibMMP1TIMP3}$	3.0E-7 mol^{-1}s^{-1}	$k_{synMKP1}$	2.5E-5 s^{-1}
$k_{degcFosmRNA}$	3.0E-3 s^{-1}	$k_{inhibMMP1TIMP3}$	1.0E-8 mol^{-1}s^{-1}	$k_{synMKP1cJun}$	1.0E-6 s^{-1}
$k_{degcJun}$	1.3E-4s^{-1}	$k_{inhibMMP3TIMP1}$	3.0E-7 mol^{-1}s^{-1}	$k_{synMMP1}$	1.5E-4 s^{-1}
$k_{degcJunmRNA}$	3.0E-3 s^{-1}	$k_{inhibMMP3TIMP3}$	1.0E-8 mol^{-1}s^{-1}	$k_{synMMP13}$	1.5E-5 s^{-1}
$k_{degCollagen2mmp1}$	5.0E-12 mol^{-1}s^{-1}	$k_{inhibTRAF6}$	0.5 mol^{-1}s^{-1}	$k_{synMMP13mRNA}$	5.0E-4 s^{-1}
$k_{degCollagen2mmp13}$	5.0E-11 mol^{-1}s^{-1}	$k_{nuc2cytSTAT3}$	1.0E-3 s^{-1}	$k_{synMMP13mRNAcJun}$	2.0E-5 s^{-1}
$k_{degDUSP16}$	1.3E-4 s^{-1}	$k_{phoscFos}$	5.0E-7 mol^{-1}s^{-1}	$k_{synMMP1mRNA}$	5.0E-3 s^{-1}
k_{degIL1}	2.0E-4 s^{-1}	$k_{phoscJun}$	1.0E-4 mol^{-1}s^{-1}	$k_{synMMP1mRNAcJun}$	2.0E-4 s^{-1}
$k_{degMKP1}$	1.0E-4 s^{-1}	$k_{phosJAK1}$	1.0E-5 mol^{-1}s^{-1}	$k_{synMMP3}$	3.0E-5 s^{-1}
$k_{degMMP1}$	1.0E-6 s^{-1}	$k_{phosJNK}$	1.0E-4 mol^{-1}s^{-1}	$k_{synMMP3mRNA}$	5.0E-3 s^{-1}
$k_{degMMP13}$	1.0E-6 s^{-1}	$k_{phosp38}$	1.0E-4 mol^{-1}s^{-1}	$k_{synMMP3mRNAcJun}$	2.0E-4 s^{-1}
$k_{degMMP13mRNA}$	6.4E-6 s^{-1}	$k_{phosSTAT3}$	5.0E-3 mol^{-1}s^{-1}	$k_{synMMPActivator}$	9.0E-10 s^{-1}
$k_{degMMP1mRNA}$	6.4E-6 s^{-1}	$k_{relADAMTS4TIMP1}$	1.0E-3 s^{-1}	k_{synPP4}	5.0E-3 s^{-1}
$k_{degMMP3}$	1.0E-6 s^{-1}	$k_{relADAMTS4TIMP3}$	1.0E-3 s^{-1}	$k_{synPP4cJun}$	2.0E-4 s^{-1}
$k_{degMMP3mRNA}$	6.4E-6 s^{-1}	$k_{relcFoscJun}$	4.0E-5 s^{-1}	$k_{synPTPRT}$	1.0E-4 s^{-1}
$k_{degMMPActivator}$	8.0E-6 s^{-1}	$k_{relL1IL1R}$	1.0E-3 s^{-1}	$k_{synSOCS3}$	1.0E-3 s^{-1}
k_{degOSM}	4.8E-5 s^{-1}	$k_{relL1IL1Ra}$	1.0E-4 s^{-1}	$k_{synSOCS3mRNA}$	6.0E-3 s^{-1}
k_{degPP4}	1.0E-4 s^{-1}	$k_{relIRAK2}$	1.0E-3 s^{-1}	k_{synSP1}	2.0E-5 s^{-1}
$k_{degPTPRT}$	5.0E-5 s^{-1}	$k_{relMMP1}$	1.0E-3 s^{-1}	$k_{synTIMP1}$	2.0E-4 s^{-1}
$k_{degSOCS3}$	8.0E-4 s^{-1}	$k_{relMMP13}$	1.0E-3 s^{-1}	$k_{synTIMP1mRNA}$	5.0E-7 mol^{-1}s^{-1}
$k_{degSOCS3mRNA}$	4.0E-4 s^{-1}	$k_{relMMP13TIMP3}$	1.0E-3 s^{-1}	$k_{synTIMP1mRNAStat3}$	4.0E-5 mol^{-1}s^{-1}
k_{degSP1}	2.0E-5 s^{-1}	$k_{relMMP1TIMP3}$	1.0E-3 s^{-1}	$k_{synTIMP3}$	4.0E-4 s^{-1}
$k_{degTIMP1}$	2.0E-5 s^{-1}	$k_{relMMP3}$	1.0E-3 s^{-1}	$k_{synTIMP3mRNA}$	5.0E-7 s^{-1}
$k_{degTIMP1mRNA}$	1.4E-5 s^{-1}	$k_{relMMP3TIMP3}$	1.0E-3 s^{-1}	$k_{synTIMP3mRNAStat3}$	4.0E-5 s^{-1}
$k_{degTIMP3}$	2.0E-5 s^{-1}	$k_{relOSMOSMR}$	1.0E-5 s^{-1}		
$k_{degTIMP3mRNA}$	1.4E-5 s^{-1}	$k_{relOSMOSMRa}$	1.0E-5s^{-1}		

[a]mol = number of molecules

Table S4 Simulated treatments

	Initial value		
Treatment	IL1	OSM	MMP Activator
No cytokines	0	0	0
IL-1 only	100	0	0
OSM only	0	100	0
IL-1 + OSM	100	100	0
IL-1+OSM+MMPActivator	100	100	100

Table S5 Simulated interventions

Intervention	Model adjustment
Inhibition of IL1 receptor by antagonist	Changed initial amount of IL1Ra
Inhibition of OSM receptor by antagonist	Changed initial amount of OSMRa
Inhibition of JAK1 activity	Varied parameter $k_{phosSTAT3}$
Inhibition of p38 activity	Varied parameter $k_{phoscFos}$
Inhibition of JNK activity	Varied parameter $k_{phoscJun}$
TIMP1 overexpression	Changed initial amount of TIMP1
TIMP3 overexpression	Changed initial amount of TIMP3

Table S6 Effect of IL1 and OSM antagonist on collagen and aggrecan release after induction by IL-1 + OSM + MMP activator

IL1Ra/IL1R ratio	OSMRa/OSMR ratio	% collagen release (day 14)	% aggrecan release (day 14)
0	0	10.2	87.5
1	0	9.0	84.0
10	0	2.9	52.4
100	0	0.1	12.3
1000	0	0.01	4.1
0	1	10.2	87.4
0	10	10.2	87.9
0	100	9.4	87.2
0	1000	7.1	80.3
1	1	8.9	83.9
10	10	3.0	53.8
100	100	0.1	11.9
1000	1000	0.01	4.6

Table S7 Experimental data for model construction

Cell type	Human T/C28a4 chondrocytes
Experimental procedure	Northern blotting of mRNA stimulated with IL-1α+ OSM. Total cellular RNA (20 µg) s was harvested at various time points after stimulation with IL-1α (1 ng/ml), OSM (10 ng/ml) , or IL-1α+OSM (1 ng/ml and 10 ng/ml, respectively) or without cytokine stimulation (control)
mRNA analysed	MMP-1, TIMP-1, TIMP-2 and GADPH
Time-points after stimulation (hours)	4, 8, 12, 24, 48, 72

Table S8 Experimental data for model validation

Cytokine added	Cell type	Measured output	Time to induction	Time of maximal induction	Time to return to basal level	Reference
IL-1β	Rabbit articular chondrocytes	Phospho-JNK	10 min	0.25-1 h	6 h	Hwang et al., 2005, J Biol Chem, 33: 29780-7
IL-1β	"	Phospho-cJun	30 min	1-3 h	> 6 h	"
OSM	Human chondrocytes from arthritic femoral head cartilage	Phospho-JAK1	5 min	15-20 min	1 h	Li et al., 2001, J of Immunol., 166: 3491-8.
OSM	"	Phospho-STAT1	5 min	15-20 min	> 1 h	"
OSM	"	Phospho-p38	5 min	15 min	> 1 h	"
